# Inheritance of brewing-relevant phenotypes in constructed *Saccharomyces cerevisiae* × *Saccharomyces eubayanus* hybrids

**DOI:** 10.1186/s12934-017-0679-8

**Published:** 2017-04-21

**Authors:** Kristoffer Krogerus, Tuulikki Seppänen-Laakso, Sandra Castillo, Brian Gibson

**Affiliations:** 1 VTT Technical Research Centre of Finland, Tietotie 2, P.O. Box 1000, 02044 Espoo, Finland; 2 Department of Biotechnology and Chemical Technology, Aalto University, School of Chemical Technology, Kemistintie 1, Aalto, P.O. Box 16100, 00076 Espoo, Finland

**Keywords:** Yeast, Beer, Rare mating, Lipid, Fatty acid, Phenolic off-flavour, Aroma

## Abstract

**Background:**

Interspecific hybridization has proven to be a potentially valuable technique for generating de novo lager yeast strains that possess diverse and improved traits compared to their parent strains. To further enhance the value of hybridization for strain development, it would be desirable to combine phenotypic traits from more than two parent strains, as well as remove unwanted traits from hybrids. One such trait, that has limited the industrial use of de novo lager yeast hybrids, is their inherent tendency to produce phenolic off-flavours; an undesirable trait inherited from the *Saccharomyces eubayanus* parent. Trait removal and the addition of traits from a third strain could be achieved through sporulation and meiotic recombination or further mating. However, interspecies hybrids tend to be sterile, which impedes this opportunity.

**Results:**

Here we generated a set of five hybrids from three different parent strains, two of which contained DNA from all three parent strains. These hybrids were constructed with fertile allotetraploid intermediates, which were capable of efficient sporulation. We used these eight brewing strains to examine two brewing-relevant phenotypes: stress tolerance and phenolic off-flavour formation. Lipidomics and multivariate analysis revealed links between several lipid species and the ability to ferment in low temperatures and high ethanol concentrations. Unsaturated fatty acids, such as oleic acid, and ergosterol were shown to positively influence growth at high ethanol concentrations. The ability to produce phenolic off-flavours was also successfully removed from one of the hybrids, Hybrid T2, through meiotic segregation. The potential application of these strains in industrial fermentations was demonstrated in wort fermentations, which revealed that the meiotic segregant Hybrid T2 not only didn’t produce any phenolic off-flavours, but also reached the highest ethanol concentration and consumed the most maltotriose.

**Conclusions:**

Our study demonstrates the possibility of constructing complex yeast hybrids that possess traits that are relevant to industrial lager beer fermentation and that are derived from several parent strains. Yeast lipid composition was also shown to have a central role in determining ethanol and cold tolerance in brewing strains.

**Electronic supplementary material:**

The online version of this article (doi:10.1186/s12934-017-0679-8) contains supplementary material, which is available to authorized users.

## Background

Yeast hybrids have been extensively used for centuries in the brewing and winemaking industries [1]. Lager yeast in particular, a natural interspecies hybrid between *Saccharomyces cerevisiae* and *Saccharomyces eubayanus*, is used for the majority of global industrial beer production. It possesses a range of desirable phenotypes that are relevant for the production of lager beer: cold tolerance, efficient use of wort sugars, and low formation of undesirable off-flavours. Recent studies have revealed that generating new lager yeast hybrids is a powerful strain-development tool, as hybrid strains have exhibited various improved traits including faster fermentation rates, more complete sugar use, and increases in aroma compound production [2–4]. Hybridization enables the combination and enhancement of phenotypic features from two different parent strains [5]. In order to further improve the potential of hybridization for strain development, it would be desirable to combine phenotypic traits from more than two parent strains, as well as remove unwanted traits from the hybrid. This could be achieved through sporulation, meiotic recombination and further mating of the hybrid. However, interspecies yeast hybrids, such as lager yeast, tend to be sterile, and therefore their sporulation efficiencies and spore viabilities are usually poor [6–9]. Studies have revealed that allotetraploid hybrids are usually not constrained by sterility [6, 8], and these tend to be capable of producing viable diploid spores. Hence, allotetraploid interspecific hybrids may undergo meiosis, during which recombination may give rise to crossovers and gene conversions [10]. This in turn causes phenotypic variation, as traits may get strengthened, weakened or even removed [11].

Many strains of *Saccharomyces* produce vinyl phenols (POF; phenolic off-flavours) from hydroxycinnamic acids, and these phenolic compounds are considered undesirable in lager beer. The most well-studied of these vinyl phenols is 4-vinyl guaiacol, which is formed from ferulic acid. The ability of brewing yeast to produce volatile phenols has been attributed to the adjacent *PAD1* and *FDC1* genes, both of which are essential for the POF+ phenotype [12]. Wild yeast strains, such as the *S. eubayanus* strains that are available for de novo lager yeast creation, tend to have functional *PAD1* and *FDC1* genes, while domesticated POF− brewing yeast have nonsense or frameshift mutations in these genes, rendering them non-functional [13–15]. As the functional genes from *S. eubayanus* are passed on to any lager hybrids created from it, these hybrids have all been afflicted with the POF+ phenotype [2–4]. However, if any non-functional alleles of *PAD1* or *FDC1* are present in the hybrid genome, it may be possible to remove the POF+ phenotype through meiotic recombination as demonstrated in studies with intraspecific *S. cerevisiae* hybrids [13, 16].

Besides the ability to produce clean flavour profiles (i.e. no off-aromas such as 4-vinyl guaiacol), one of the main traits of lager yeast is its ability to stay metabolically active and ferment in the cold [1]. Recent studies have revealed that the *S. eubayanus* parent strain contributes this cold tolerance to lager yeast [4, 17]. However, unlike the POF+ phenotype, the mechanisms that contribute to cold tolerance in brewing yeast are not fully understood. It has been revealed that low temperature affects protein translation and folding efficiencies, mRNA stabilities and the product activity and expression of central metabolic genes [18–24]. Recent studies have also suggested that differences in the lipid composition of the plasma membrane play a vital role in temperature tolerance [25–27]. The fluidity of the plasma membrane is affected by its lipid composition and the temperature [26, 28], and a decrease in membrane fluidity caused by a low fermentation temperature can, in turn, result in impaired transporter function and lower nutrient uptake [27, 29]. Relatively high levels of ergosterol and low levels of unsaturated fatty acids could, for example, result in a greater tendency of cell membranes to freeze at lower temperatures, thereby reducing functionality [26, 30, 31]. The lipid composition of yeast has also been shown to influence ethanol tolerance [32]. Similarly to cold tolerance, ethanol tolerance has been shown to be dependent on unsaturated fatty acid and ergosterol concentrations [31, 33]. They have been hypothesized to function by maintaining optimum membrane thickness and fluidity by counteracting the fluidizing effects of ethanol. Here, we wanted to investigate what lipid species correlated with good fermentation performance at low temperatures and high alcohol levels, by examining how the lipidomes of brewing yeasts react to changes in temperature and ethanol concentration.

In this study we wanted to both demonstrate the possibility of using an allotetraploid interspecific hybrid as an intermediate to create a POF− lager hybrid with DNA from three parent strains, and use these hybrids to elucidate relationships between different lipid species and tolerance towards low temperatures and high ethanol concentrations. This was accomplished by first rare mating a set of three parent strains, all with different desirable properties, to produce five different hybrid strains. Their fermentation kinetics and lipid compositions were compared in small-scale fermentations performed at low temperatures and in the presence of ethanol. Lipids were analysed using both a targeted GC/MS and non-targeted UPLC/MS approach. Using multivariate analysis, it was revealed that high levels of unsaturated fatty acids and especially phospholipids containing palmitoleic and oleic acid were associated with good fermentation performance at low temperatures and high ethanol concentrations. Furthermore, ergosterol was associated with good fermentation performance at high ethanol concentrations, but had a negative effect at low temperatures. These observations were supported with growth data of laboratory strains lacking *OLE1* and *ERG4* genes in media containing ethanol and oleic acid. The potential application of these strains in industrial fermentations was finally demonstrated in 2-L wort fermentations. These revealed that Hybrid T2, a meiotic segregant of Hybrid T1 (containing DNA from all three parent strains), reached the highest ethanol concentration, consumed the most maltotriose, and did not produce any 4-vinyl guaiacol, therefore making it a suitable candidate for industrial lager beer fermentations.

## Results

### Generation of inter- and intra-specific hybrids

**Fig. 1:**
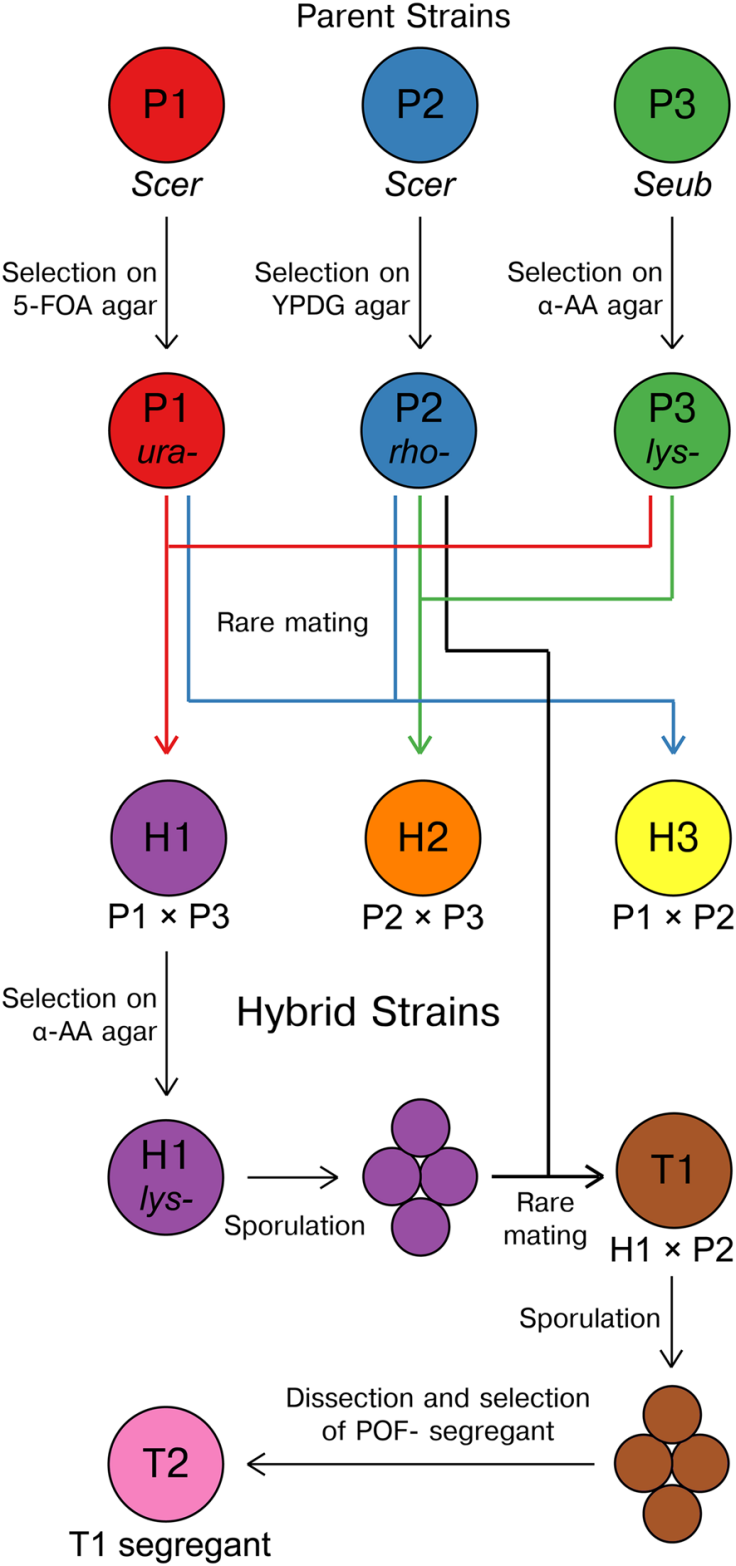
Overview of the 8 brewing yeast strains created and used in the study. First, mutants of parent strains P1–P3 carrying selection markers were selected. Hybrids H1–H3 were then obtained by rare mating parent strains P1–P3. Hybrid T1, containing DNA from all three parent strains, was obtained by sporulating the allotetraploid Hybrid H1 and rare mating dissected spores with parent P2. The POF− segregant Hybrid T2 was obtained by sporulating Hybrid T1 and screening spore clones for the absence of 4-vinyl guaiacol production. For more information see the “Methods” and Table 1. *Scer*: *Saccharomyces cerevisiae*, *Seub: Saccharomyces eubayanus*

The set of 8 brewing strains that were used in this study consisted of 3 parent strains and 5 hybrid strains (Table 1). The three parent strains, P1 and P2 being industrial ale strains and P3 being the type strain of *S. eubayanus*, were chosen for their varying phenotypic properties. Of the three, P1 is the only strain that is able to use maltotriose during fermentation, P2 is the only strain that does not produce 4-vinyl guaiacol (i.e. it is POF−), while P3 is the cold-tolerant parent strain of lager yeast. From the three parent strains, the three hybrid strains H1–H3 were first successfully constructed through rare mating according to the schematic in Fig. 1. The hybrid status of these strains was confirmed using PCR (ITS, species-specific, and interdelta primers), which showed that they contained DNA from all their parent strains (Additional file 1: Figure S1). Flow cytometry also revealed that these hybrid strains were all polyploid (Additional file 1: Figure S2), containing higher amounts of DNA than any of the 3 parent strains P1–P3.

**Table 1:** Yeast strains used in the study

ID	Species	Information	Source
Brewing strains			
P1	*S. cerevisiae*	VTT-A81062. Maltotriose fermentation, POF+	VTT culture collection
P2	*S. cerevisiae*	WLP099. No maltotriose fermentation, POF−	White Labs Inc
P3	*S. eubayanus*	VTT-C12902. No maltotriose fermentation, POF+	VTT culture collection
H1	Hybrid	P1 × P3 interspecific hybrid (VTT-A15225)	Created in this study
H2	Hybrid	P2 × P3 interspecific hybrid	Created in this study
H3	Hybrid	P1 × P2 intraspecific hybrid	Created in this study
T1	Hybrid	H1 × P2 interspecific triple hybrid	Created in this study
T2	Hybrid	Meiotic segregant of T1	Created in this study
Laboratory strains			
WT	*S. cerevisiae*	BY4741 *MAT* **a**; *his3*∆*1*; *leu2*∆*0*; *met15*∆*0*; *ura3*∆*0*	EUROSCARF (Y00000)
* ole1*∆	*S. cerevisiae*	BY4741 *MAT* **a**; *his3*∆*1*; *leu2*∆*0*; *met15*∆*0*; *ura3*∆*0*; *ole1*-*m2*:*kanMX*	EUROSCARF (Y40963)
* erg4*∆	*S. cerevisiae*	BY4741 *MAT* **a**; *his3*∆*1*; *leu2*∆*0*; *met15*∆*0*; *ura3*∆*0*; *YGL012w*::*kanMX4*	EUROSCARF (Y04380)

The tetraploid nature of H1 allowed it to form viable spores (47% viability) despite being an interspecific hybrid, and these spores could subsequently be mated with P2 to form the triple hybrid T1 (Fig. 1). PCR was again used to confirm that the strain contained DNA from all three parent strains (Additional file 1: Figure S1). Whole genome sequencing of the strains also revealed that Hybrid T1 contained a higher ratio of *S. cerevisiae*-derived to *S. eubayanus*-derived chromosomes (approximately 3:1; Additional file 1: Figure S3E). Additionally, the strain appeared to contain a chimeric chromosome consisting of approximately 355 kbp of the *S. cerevisiae* P1-derived chromosome II and 437 kbp of the *S. eubayanus* P3-derived chromosome IV, apparently formed during sporulation of Hybrid H1. Like Hybrid H1, Hybrid T1 was also able to form viable spores (38% viability). The spore clones of T1 were screened for the POF phenotype in media containing ferulic acid, and out of 12 spores clones that were assayed, only 3 were POF−. The best growing of these was given the name Hybrid T2, i.e. a POF− meiotic segregant of Hybrid T1. Whole genome sequencing of the strains revealed that Hybrid T2 contained at least one copy of the *S. cerevisiae*-derived chromosomes, but had lost several chromosomes derived from *S. eubayanus* (Additional file 1: Figure S3F).

### Effects of temperature and ethanol on fermentation rate and lipid composition

**Fig. 2:**
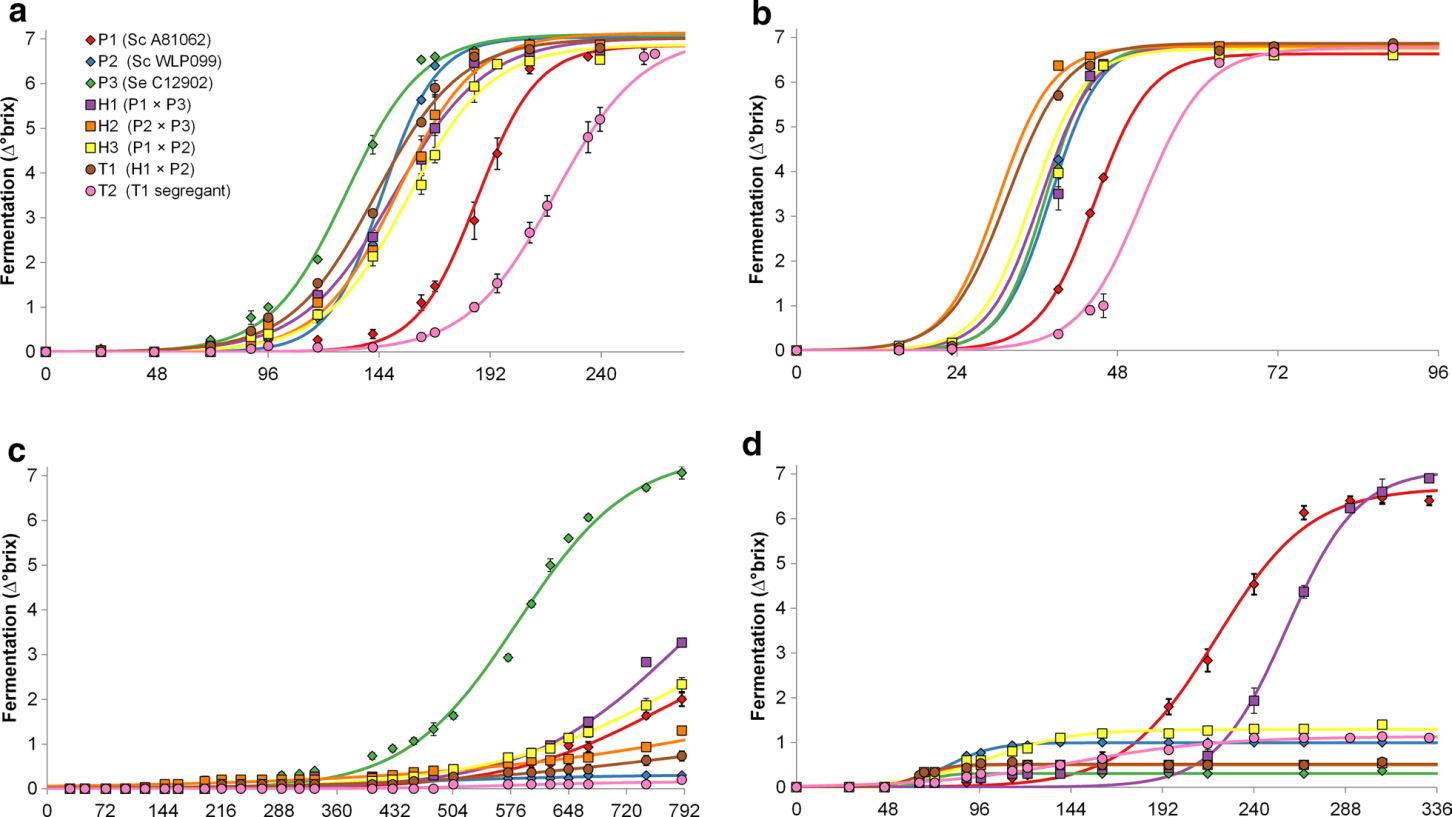
Fermentation profiles (decrease of °Brix over time as hours) of the 8 brewing strains in the growth assays performed at **a** 10 °C with no supplemented ethanol, **b** 20 °C with no supplemented ethanol, **c** 10 °C with 8% (v/v) supplemented ethanol, and **d** 20 °C with 8% (v/v) supplemented ethanol. Points represent means of measured values, with error bars showing the standard deviation of three replicate cultivations. The growth curves were modelled using the logistic model in the ‘grofit’ package for R [60]

To investigate how the fermentation rate and the lipid composition of the 8 brewing strains was affected by the fermentation temperature and ethanol content of the growth media, a set of 100 mL flask fermentations were carried out. The fermentations were performed at two temperatures, 10 and 20 °C, and with two initial ethanol concentrations, 0 and 8% (v/v). All eight strains were able to finish fermentation (a decrease in approx. 7 °brix) at both 10 and 20 °C in the media without any added ethanol (Fig. 2a, b; Table 2). While, *S. eubayanus* (P3) had the shortest lag time (λ) at 10 °C as we had expected, *S. cerevisiae* P1 and P2 rather surprisingly showed the highest maximum fermentation rate (μ) at this temperature. Despite the fast fermentation rate, the amount of biomass produced at 10 °C without any added ethanol by *S. cerevisiae* P1 and P2, and the intraspecific Hybrid H3 all showed the lowest level of accumulated biomass (*p* < 0.05) when compared to the fermentations at 20 °C without any added ethanol.

**Table 2:** Modelled (*A*, μ, λ) and measured (dry mass) fermentation and growth parameters of the fermentation assays that were performed at different temperatures (10 and 20 °C) and supplemented ethanol levels (0 and 8% (v/v) EtOH)

Strain and condition		*A* (°brix)	μ (°brix h^−1^)	λ (hours)	Dry mass (g L^−1^)
10 °C, 0% EtOH	P1	6.85 (±0.18)^ab^	0.132 (±0.0107)^ab^	160 (±2.3)^b^	9.2 (±0.65)^cd^
	P2	6.63 (±0.13)^b^	0.140 (±0.0172)^a^	122 (±2.3)^c^	9.8 (±0.05)^bcd^
	P3	7.10 (±0.15)^a^	0.108 (±0.0064)^bc^	97 (±2.2)^e^	11.0 (±0.33)^abc^
	H1 (P1 × P3)	7.02 (±0.18)^ab^	0.089 (±0.0059)^c^	111 (±3.1)^d^	12.2 (±1.62)^ab^
	H2 (P2 × P3)	7.13 (±0.19)^a^	0.106 (±0.0083)^c^	118 (±3.2)^c^	12.6 (±0.14)^a^
	H3 (P1 × P2)	6.87 (±0.16)^ab^	0.095 (±0.0058)^c^	120 (±2.6)^c^	7.5 (±1.75)^d^
	T1 (H1 × P2)	7.01 (±0.14)^ab^	0.095 (±0.0054)^c^	106 (±2.5)^d^	12.7 (±0.39)^a^
	T2 (T1 segregant)	7.05 (±0.13)^ab^	0.093 (±0.0015)^c^	182 (±0.7)^a^	10.9 (±0.16)^abc^
20 °C, 0% EtOH	P1	6.63 (±0.02)^b^	0.403 (±0.0069)^a^	36 (±0.1)^b^	12.5 (±3.12)^a^
	P2	6.86 (±0.05)^a^	0.470 (±0.0630)^a^	31 (±0.6)^c^	11.6 (±0.16)^a^
	P3	6.83 (±0.07)^a^	0.501 (±0.1218)^a^	30 (±0.8)^cd^	10.6 (±0.58)^a^
	H1 (P1 × P3)	6.83 (±0.04)^a^	0.435 (±0.0406)^a^	29 (±0.3)^de^	13.9 (±1.55)^a^
	H2 (P2 × P3)	6.80 (±0.03)^a^	0.426 (±0.0470)^a^	22 (±0.7)^f^	10.6 (±0.18)^a^
	H3 (P1 × P2)	6.74 (±0.06)^ab^	0.428 (±0.1647)^a^	27 (±0.7)^e^	13.3 (±1.88)^a^
	T1 (H1 × P2)	6.87 (±0.05)^a^	0.392 (±0.0494)^a^	23 (±1.0)^f^	13.1 (±0.80)^a^
	T2 (T1 segregant)	6.77 (±0.08)^ab^	0.357 (±0.0292)^a^	42 (±0.4)^a^	11.5 (±0.22)^a^
10 °C, 8% EtOH	P1	3.45 (±0.53)^d^	0.009 (±0.0007)^bc^	569 (±14.9)^ab^	4.3 (±0.4)^bc^
	P2	0.32 (±0.03)^e^	0.001 (±0.0001)^d^	186 (±28.3)^d^	0.3 (±0.04)^bc^
	P3	7.50 (±0.20)^a^	0.027 (±0.0010)^a^	446 (±5.7)^c^	15.7 (±1.97)^a^
	H1 (P1 × P3)	6.45 (±0.96)^ab^	0.017 (±0.0082)^b^	596 (±58.9)^a^	8.1 (±5.26)^b^
	H2 (P2 × P3)	4.22 (±0.63)^cd^	0.004 (±0.0008)^cd^	559 (±43.5)^ab^	6.4 (±0.24)^b^
	H3 (P1 × P2)	5.14 (±0.60)^bc^	0.010 (±0.0008)^bc^	561 (±16.6)^ab^	3.3 (±0.09)^bc^
	T1 (H1 × P2)	1.53 (±0.51)^e^	0.002 (±0.0005)^cd^	464 (±66.3)^bc^	3.3 (±0.70)^bc^
	T2 (T1 segregant)	NA	NA	NA	NA
20 °C, 8% EtOH	P1	6.69 (±0.11)^b^	0.068 (±0.0036)^b^	171 (±2.8)^b^	7.5 (±1.53)^b^
	P2	1.00 (±0.01)^d^	0.020 (±0.0011)^c^	56 (±1.3)^d^	1.5 (±0.08)^c^
	P3	0.31 (±0.01)^e^	0.013 (±0.0031)^cd^	57 (±2.9)^d^	0.8 (±0.10)^c^
	H1 (P1 × P3)	7.09 (±0.19)^a^	0.095 (±0.0068)^a^	220 (±2.9)^a^	14.9 (±0.10)^a^
	H2 (P2 × P3)	0.50 (±0.01)^e^	0.015 (±0.0033)^cd^	49 (±4.1)^d^	1.5 (±0.06)^c^
	H3 (P1 × P2)	1.29 (±0.03)^c^	0.015 (±0.0014)^cd^	58 (±4.4)^d^	1.5 (±0.09)^c^
	T1 (H1 × P2)	0.51 (±0.01)^e^	0.016 (±0.0037)^cd^	49 (±4.4)^d^	1.1 (±0.06)^c^
	T2 (T1 segregant)	1.14 (±0.03)^cd^	0.007 (±0.0005)^d^	70 (±4.5)^c^	1.3 (±0.04)^c^

*NA* not available

The growth curves were modelled using the logistic model in the ‘grofit’ package for R. Values were determined from three independent cultivations (standard deviation in parentheses). Values in the same group in the same column with different superscript letters (a–f) differ significantly (*p* < 0.05 as determined with one-way ANOVA with Tukey’s post hoc test)

Fermentations were not as efficient in the growth media supplemented with 8% (v/v) ethanol. At 10 °C, it was only *S. eubayanus* P3 and Hybrid H1 which managed to reach the same maximum fermentation level (A) as in the unsupplemented growth media. However, at 20 °C P3 both fermented and grew the worst of the 8 strains, suggesting it is ethanol-tolerant at 10 °C but not 20 °C. *S. cerevisiae* P1 on the other hand, performed relatively well in the presence of ethanol at 20 °C, but not at 10 °C. The interspecific hybrid between the two, Hybrid H1, was able to ferment in the presence of ethanol at both 10 and 20 °C, and even outperforming P1 with regards to maximum fermentation rate (μ) at 20 °C. The two triple hybrids T1 and T2, as well as the *S. cerevisiae* P2 (the genome of which dominates in T1 and T2), performed poorly in the presence of ethanol at both 10 and 20 °C. Hybrid T2 did not grow or ferment at 10 °C and with 8% supplemented ethanol, and samples of it at this condition were thus excluded from subsequent lipid analysis.

**Fig. 3:**
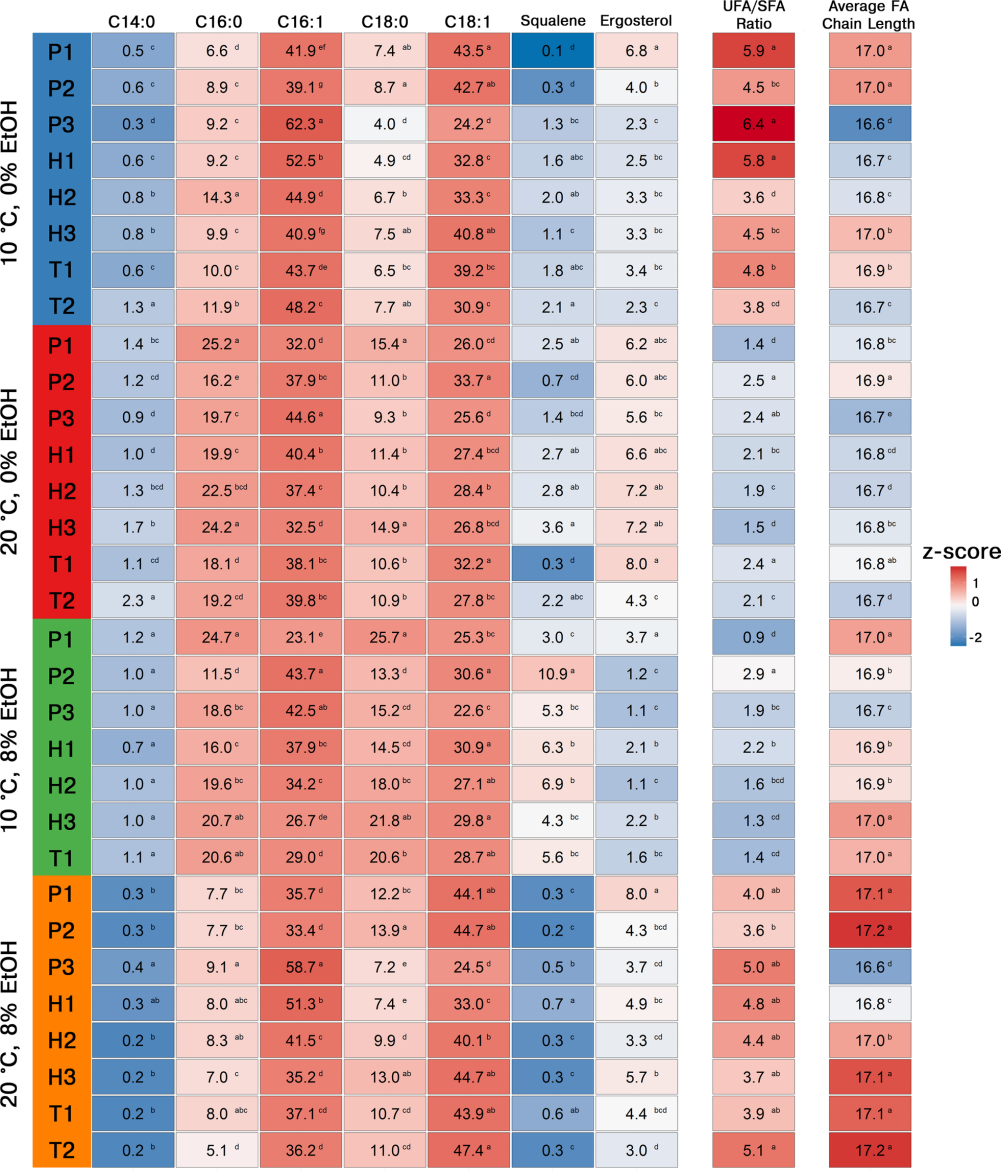
The relative concentrations (% of total lipid content) of fatty acids, squalene and ergosterol, unsaturated (UFA) to saturated (SFA) fatty acid ratio, and average fatty acid (FA) chain length in the lipids extracted from cells in late exponential phase during the growth assays. Values are means from three independent cultivations. Values in the same group in the same column with different superscript letters (*a*–*g*) differ significantly (*p* < 0.05 as determined with one-way ANOVA with Tukey’s post hoc test). The heatmap was generated based on the z-scores (a *blue color* indicates a negative z-score and lower concentration, while a *red color* indicates a positive z-score and a higher concentration)

Analysis of the fatty acid composition, squalene content, and ergosterol content of the 8 brewing yeast strains sampled at the end of the exponential phase during the flask fermentations revealed significant differences between the different strains and conditions (Fig. 3; Additional file 1: Table S1). In almost all samples, the unsaturated fatty acids palmitoleic acid (C16:1) and oleic acid (C18:1) were the most prevalent of the fatty acids. Of the strains, *S. eubayanus* P3 was particularly high in palmitoleic acid content, while *S. cerevisiae* P2 was particularly high in oleic acid content. When conditions were changed from the control (20 °C and no supplemented ethanol), there was a slight increase in both the ratio of unsaturated to saturated fatty acids (from 2.0 up to 4.9) and average chain length (from 16.8 up to 17.0). An exception was the fermentations at 10 °C and 8% supplemented ethanol, where the ratio of unsaturated to saturated fatty acids instead decreased slightly (from 2.0 to 1.7). The concentrations of ergosterol were in general higher at the fermentations performed at 20 °C compared to those at 10 °C. Of the strains, *S. cerevisiae* P1 tended to have the highest levels of ergosterol, while *S. eubayanus* P3 tended to have the lowest levels of ergosterol. No clear patterns were observed between the strains and the conditions for the squalene concentrations, other than that the highest concentrations of squalene were measured for the fermentations performed at 10 °C and 8% supplemented ethanol.

**Fig. 4:**
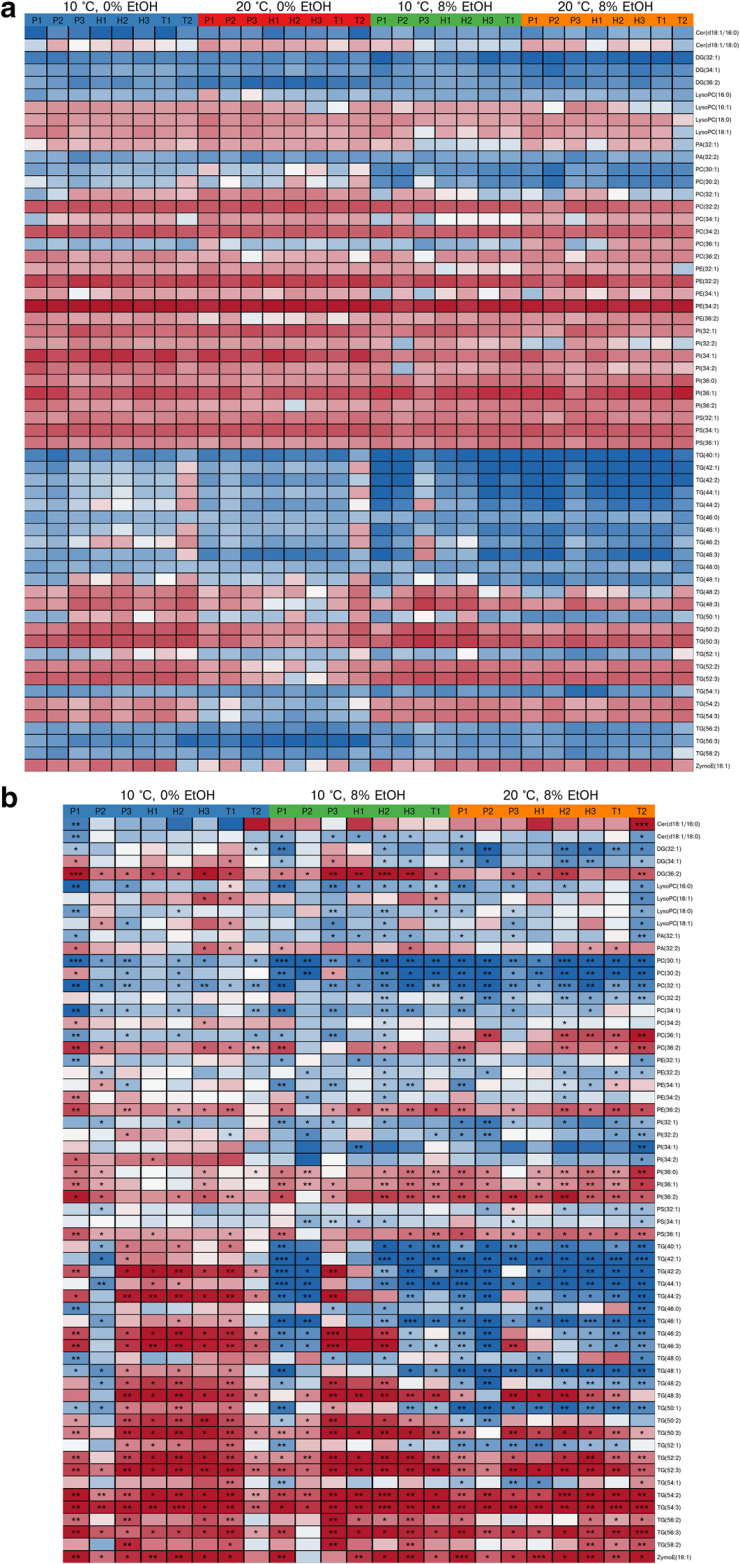
The **a** relative log10-transformed concentrations (% of total lipid content), and **b** log2 fold change (in comparison to the concentrations measured for the cultivations performed at 20 °C and 0% supplemented EtOH) of the relative concentrations (% of total lipid content), of the 60 lipid species determined by UPLC/MS lipidomics in the lipids extracted from cells in late exponential phase during the growth assays. Values are means from three independent cultivations. **a** The heatmap was generated based on the z-scores (a *blue color* indicates a negative z-score and lower concentration, while a *red color* indicates a positive z-score and a higher concentration). **b** A *blue color* indicates a decrease in concentration, while a *red color* indicates an increase in concentration compared to the cultivation at 20 °C and 0% supplemented EtOH. The *asterisks* indicate whether the change was significant as determined by Student’s t test (unpaired, two-tailed and unequal variance) corrected with the Benjamini-Hochberg procedure (*p < 0.05; **p < 0.01; ***p < 0.001)

The UPLC/MS lipidomics analysis of the 8 brewing yeast strains sampled at the end of the exponential phase during the flask fermentations also revealed differences between the different strains and conditions (Fig. 4). Based on retention times and m/z spectra we identified 60 lipid species from the samples (Additional file 2). The most prevalent lipid groups in the samples were the phosphatidylethanolamines (PE) and phosphatidylinositols (PI). Of the individual lipid species, PE(32:2), PE(34:2), PI(34:1), PI(34:2) and PI(36:1) were present in the highest concentrations (Fig. 4a). As the growth temperature was lowered from 20 to 10 °C and ethanol was supplemented to the growth media, significant changes were observed for 55% of the lipid species (Fig. 4b). Similarly to what was revealed by the fatty acid analysis, there was an increase in unsaturation ratio and chain length among the lipid species when the temperature was lowered and the ethanol content was increased.

**Fig. 5:**
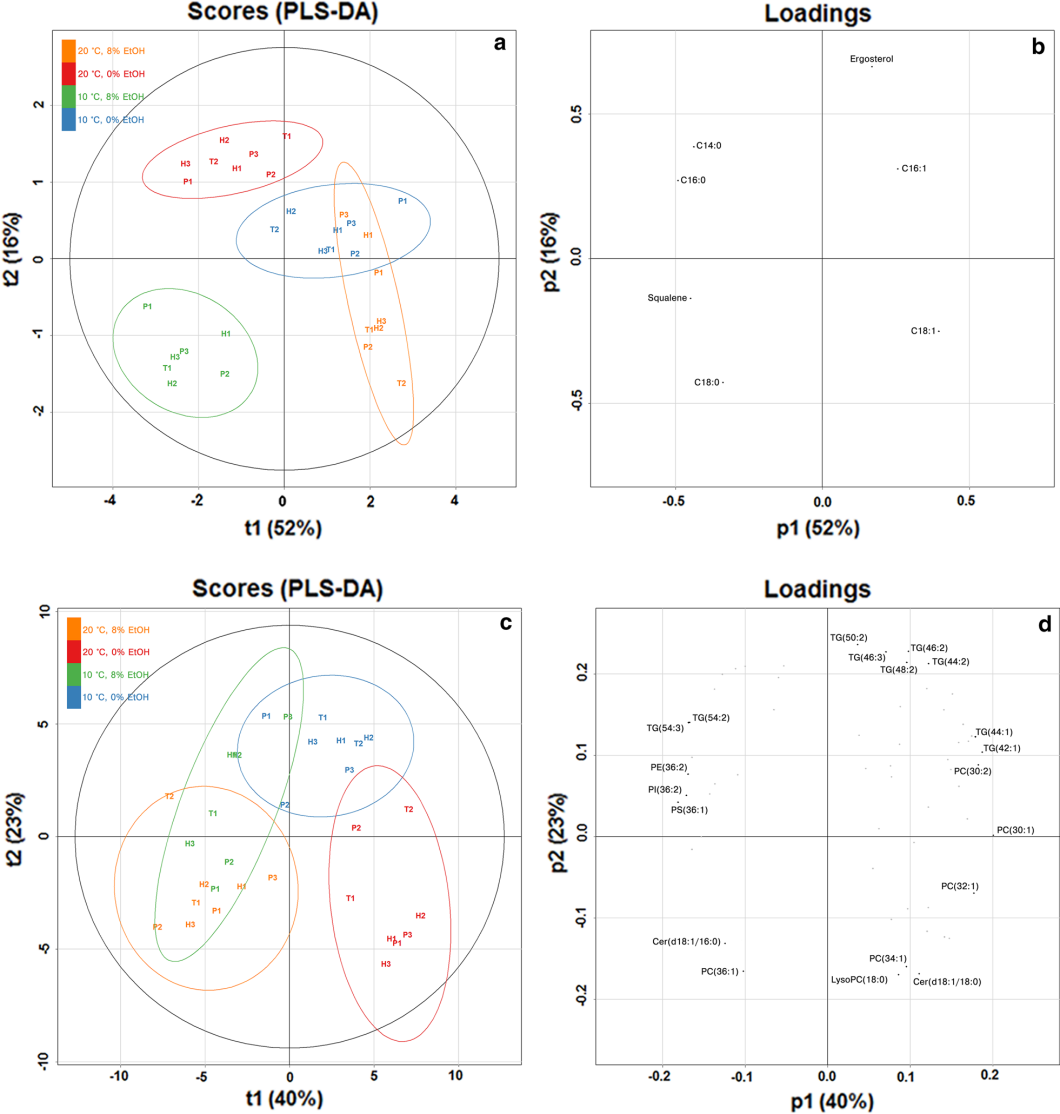
Score and loading plots of the PLS-DA models built on **a**, **b** the fatty acid, squalene and ergosterol compositions obtained from GC/MS analysis, and **c**, **d** the compositions of the 60 lipid species obtained from UPLC/MS lipidomics analysis. Model properties: **a**, **b** *R* ^2^ = 0.69, *Q* ^2^ = 0.59 (*p* < 0.05 by random permutation test), and model uses 3 components; **c**, **d** *R* ^2^ = 0.64, *Q* ^2^ = 0.54 (*p* < 0.05 by random permutation test), and model uses 3 components

Using Partial Least Squares Discriminant Analysis (PLS-DA), it was possible to separate the four different fermentation conditions based on the lipid data. A PLS-DA model was first constructed based on the measured fatty acid, squalene and ergosterol compositions (Fig. 5a, b). The PLS-DA model was cross-validated and can be considered significant (*Q* ^2^ > 0.5) [34]. As indicated by the compositions in Fig. 3, there is considerable overlap between the fermentations at 10 °C and no supplemented ethanol and 20 °C with 8% supplemented in the PLS-DA model, suggesting that the fatty acid composition of these strains respond similarly in regards to cold and ethanol stress. A cross-validated and significant PLS-DA model could also separate the four different fermentation conditions based on the compositions of the 60 lipid species determined by UPLC/MS (Fig. 5c, d). The four groups were separated by initial ethanol content along the first model component (t1) and the temperature along the second model component (t2). Like the PLS-DA model produced from the fatty acid, squalene and ergosterol data (Fig. 5a, b), there was considerable overlap between the lipid profiles of the fermentations at lower temperature and with supplemented ethanol, again suggesting that the lipid composition of these strains respond similarly in regards to cold and ethanol stress.

**Fig. 6:**
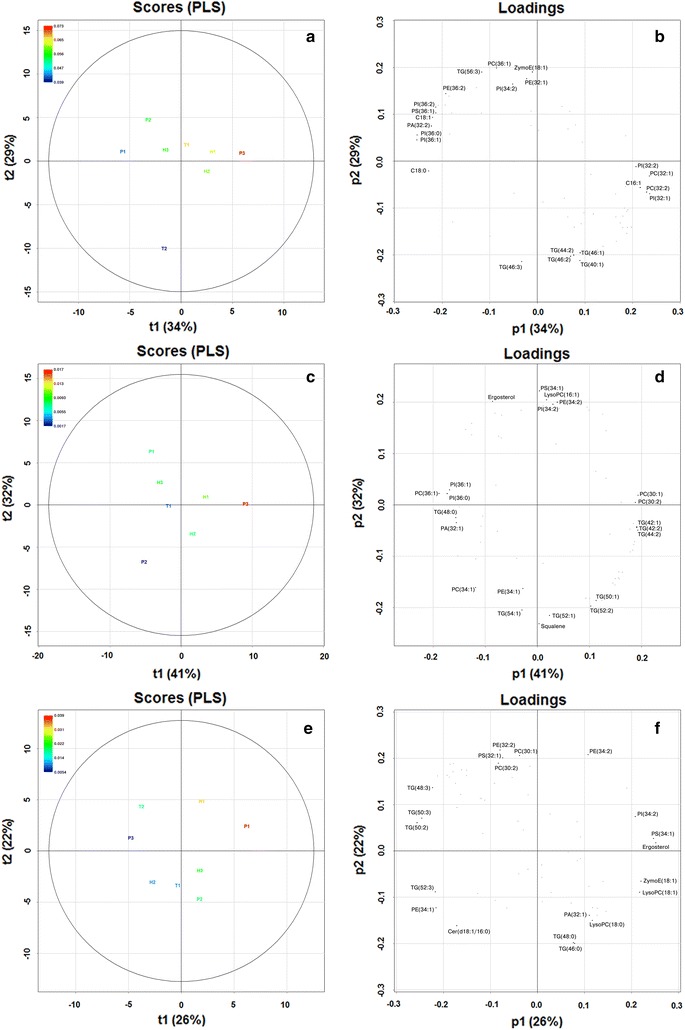
Score and loading plots of the PLS models built from the fermentation and lipid data obtained at different temperatures and supplemented ethanol levels (**a**, **b** 10 °C, 0% EtOH; **c**, **d** 10 °C, 8% EtOH; **e**, **f** 20 °C, 8% EtOH). The *Y* variable of the models was the maximum fermentation level divided by the lag time (*A* and λ from Table 2, respectively), while the *X* variables were the combined dataset of the compositions of fatty acids, squalene and ergosterol obtained from GC/MS analysis and the compositions of the 60 lipid species obtained from UPLC/MS lipidomics analysis. Model properties: **a**, **b** *R* ^2^ = 0.95, *Q* ^2^ = 0.76 (*p* < 0.05 by random permutation test), and model uses 2 components; **c**, **d** *R* ^2^ = 0.87, *Q* ^2^ = 0.49 (*p* < 0.05 by random permutation test), and model uses 2 components; **e**, **f** *R* ^2^ = 0.97, *Q* ^2^ = 0.51 (*p* < 0.05 by random permutation test), and model uses 2 components

In order to elucidate what lipid species were associated with the yeast strains showing good fermentation performance at 10 °C and in the presence of ethanol, multivariate analysis was performed on both the lipid data (GC/MS and UPLC/MS) and the fermentation parameters (Table 2). Fermentation performance was quantitated based on the ratio (A/λ) of the maximum fermentation level (A in Table 2) and the lag time (λ in Table 2). Hence, this value increases with an increased fermentation level or a shorter lag time. It was attempted to construct PLS regression models for the different fermentation conditions using this ratio (A/λ) as the *Y* response variable and the lipid data as the *X* predictor variables (Fig. 6). Only the models produced using the data from the fermentation at 10 °C and no supplemented ethanol (Fig. 6a, b) and 20 °C and 8% supplemented ethanol (Fig. 6e, f) were successfully cross-validated (Q^2^ > 0.5 and significant at *p* < 0.05 by random permutation test [34]). The model produced using the data from the fermentation at 10 °C and 8% supplemented ethanol (Fig. 6c, d) had a Q^2^ value of 0.49 (*p* < 0.05), i.e. just below the threshold of 0.5, and will still be presented.

The three models all showed an increase in fermentation performance along the first model component (t1), with the highest *Y* values (i.e. A/λ) in the upper right quadrant (Fig. 6a, c, e). The 10 individual lipid species which had the largest Variable Importance in Projection (VIP) scores and regression coefficients for each PLS model are shown in Table 3. These are the lipid species that had either the largest positive or negative effect on the *Y* response variable, i.e. the fermentation performance. At 10 °C and no supplemented ethanol, phosphatidylcholines (PC), phosphatidylinositols (PI) and sterol esters containing unsaturated fatty acids seem to positively correlate with fermentation performance, while longer-chain phosphatidylinositols and phosphatidylserines (e.g. PI(36:0), PI(36:1), PI(36:2) and PS(36:1)) and saturated fatty acids (e.g. C14:0 and C18:0) were negatively correlated with fermentation performance (Fig. 6a, b; Table 3). Similarly at 10 °C and 8% supplemented ethanol, phosphatidylcholines (PC) and phosphatidylethanolamines (PE) containing palmitoleic acid [e.g. PC(30:1), PC(30:2) and PE(32:2)] seem to positively correlate with fermentation performance, while longer-chain phosphatidylcholines and phosphatidylethanolamines [e.g. PC(34:1), PC(36:1), and PE(34:1)] were negatively correlated with fermentation performance (Fig. 6c, d; Table 3). Finally, at 20 °C and 8% supplemented ethanol, phosphatidylethanolamines (PE), phosphatidylinositols (PI), and phosphatidylserines (PS) containing either or both oleic and palmitoleic acid [e.g. PE(34:2), PI(34:1), PI(34:2), and PS(34:1)] seem to positively correlate with fermentation performance (Fig. 6e, f; Table 3). Ergosterol was also positively correlated with fermentation performance. These results would suggest that good fermentation performance at both lower temperatures and in higher ethanol concentrations are associated with increased amounts of unsaturated fatty acids in the yeast. Increased ergosterol concentrations seem to have a positive effect on fermentation performance in higher ethanol concentrations, but this effect is negated at lower temperatures.

**Table 3:** The 10 most significant Variable Importance in Projection (VIP) scores and regressions coefficients of the three PLS models presented in Fig. 6

10 °C, 0% supplemented EtOH				10 °C, 8% supplemented EtOH				20 °C, 8% supplemented EtOH			
VIP scores		Regression coefficients		VIP scores		Regression coefficients		VIP scores		Regression coefficients	
PC(30:2)	1.99	PC(30:2)	−0.1029	PC(30:1)	1.62	TG(58:2)	0.0524	PE(34:1)	2.01	Cer(d18:1/16:0)	−0.1003
PC(34:1)	1.71	TG(46:3)	−0.0882	TG(58:2)	1.58	TG(56:3)	0.0477	Cer(d18:1/16:0)	1.99	PI(34:1)	0.0967
TG(46:3)	1.66	C14:0 (FA)	−0.0876	PC(30:2)	1.47	PC(30:1)	0.0450	PI(34:1)	1.96	PE(34:1)	−0.0963
C14:0 (FA)	1.65	PC(34:1)	0.0859	TG(56:3)	1.45	TG(40:1)	0.0421	TG(52:3)	1.87	PE(34:2)	0.0963
C18:0 (FA)	1.60	C18:0 (FA)	−0.0741	TG(40:1)	1.44	PC(34:1)	−0.0418	PE(34:2)	1.83	TG(52:3)	−0.0869
PC(32:1)	1.49	ZymoE(18:1)	0.0726	TG(56:2)	1.42	PA(32:2)	−0.0416	PS(34:1)	1.80	PI(34:2)	0.0779
PI(36:1)	1.47	PC(32:1)	0.0652	PA(32:2)	1.40	PE(32:2)	0.0399	Ergosterol	1.80	PS(34:1)	0.0765
PI(36:0)	1.41	PI(36:1)	−0.0596	TG(44:2)	1.38	TG(54:3)	0.0397	PI(34:2)	1.71	Ergosterol	0.0752
ZymoE(18:1)	1.39	PI(34:1)	0.0587	TG(42:1)	1.38	PE(34:1)	−0.0397	TG(50:2)	1.58	TG(50:3)	−0.0566
PC(32:2)	1.30	PE(32:1)	0.0581	PC(36:1)	1.38	PC(30:2)	0.0381	TG(50:3)	1.55	TG(50:2)	−0.0559

### Supplementation of oleic acid enhances growth in the presence of ethanol

**Fig. 7:**
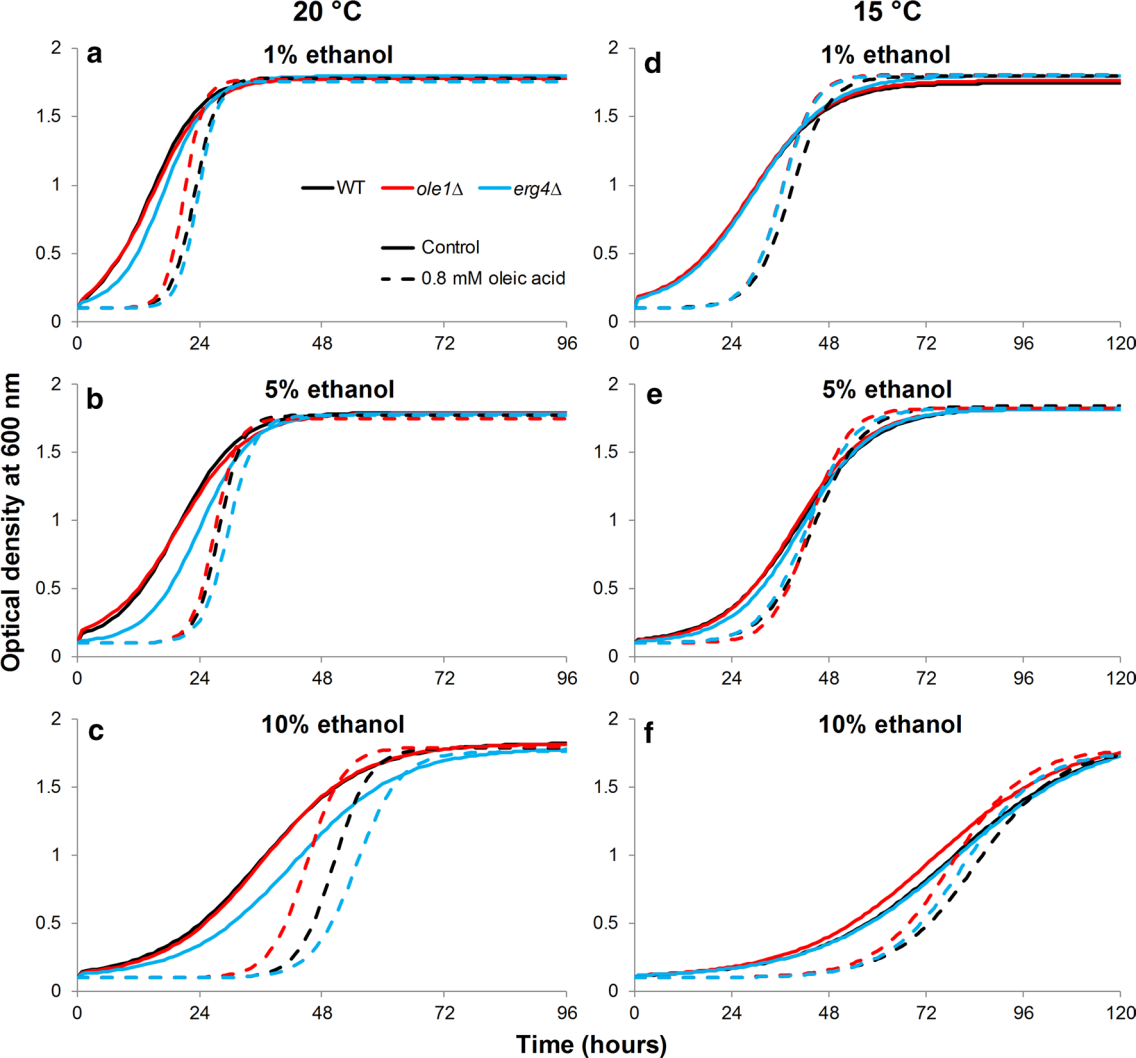
The growth curves (OD600) of the three laboratory strains grown in media supplemented with 0.8 mM oleic acid (*dashed line*) and **a**, **d** 1%, **b**, **e** 5%, or **c**, **f** 10% ethanol. Cultivations were performed in microplate format at 20 °C (**a**–**c**) and 15 °C (**d**–**f**). The growth curves were modelled using the logistic model in the ‘grofit’ package for R [60] based on data obtained from four biological replicates during microplate cultivations. Model parameters are presented in Additional file 1: Table S2

It has been shown that *Saccharomyces* strains are able to incorporate exogenous fatty acids from the growth media and these fatty acids can constitute a considerable fraction of the total fatty acids in the cell [35]. To test the effects that ergosterol and unsaturated fatty acids have on the ability to grow in the presence of ethanol and at sub-optimal temperatures, microplate cultivations of a laboratory strain WT and two knockout strains, *ole1*∆ and *erg4*∆, unable to synthesize unsaturated fatty acids and ergosterol respectively (Table 1), were carried out in media containing varying concentrations of supplemented ethanol (1, 5 or 10%) and oleic acid (0 or 0.8 mM) at two different temperatures (20 and 15 °C). At 20 °C in 1% ethanol and no added oleic acid, the three strains performed similarly (Fig. 7a; Additional file 1: Table S2). As the ethanol concentration was increased to 5 and 10%, the *erg4*∆ strain grew slightly slower and had longer lag times than the other strains, suggesting that ergosterol enhances ethanol tolerance (Fig. 7b, c; Additional file 1: Table S2). At 15 °C, this effect wasn’t observed, as the *erg4*∆ strain performed similarly to the WT strain when no oleic acid was added. While ergosterol may enhance ethanol tolerance, results suggest that it may decrease the ability to grow in sub-optimal temperatures. The addition of oleic acid to the growth media resulted in longer lag times for all strains in all conditions (Fig. 7; Additional file 1: Table S2). However, the presence of oleic acid considerably increased the growth rate of all the strains. This was particularly apparent at 20 °C and 10% ethanol, as well as at 15 °C with 1 and 5% ethanol, where e.g. the *ole1*∆ strain reached the maximum OD600 level several hours earlier when grown in the presence of oleic acid (Fig. 7c–e; Additional file 1: Table S2). This suggests that exogenous oleic acid enhances growth when ethanol concentration is increased in the media and at sub-optimal growth temperatures.

### Fermentations in wort confirm POF− phenotype

**Fig. 8:**
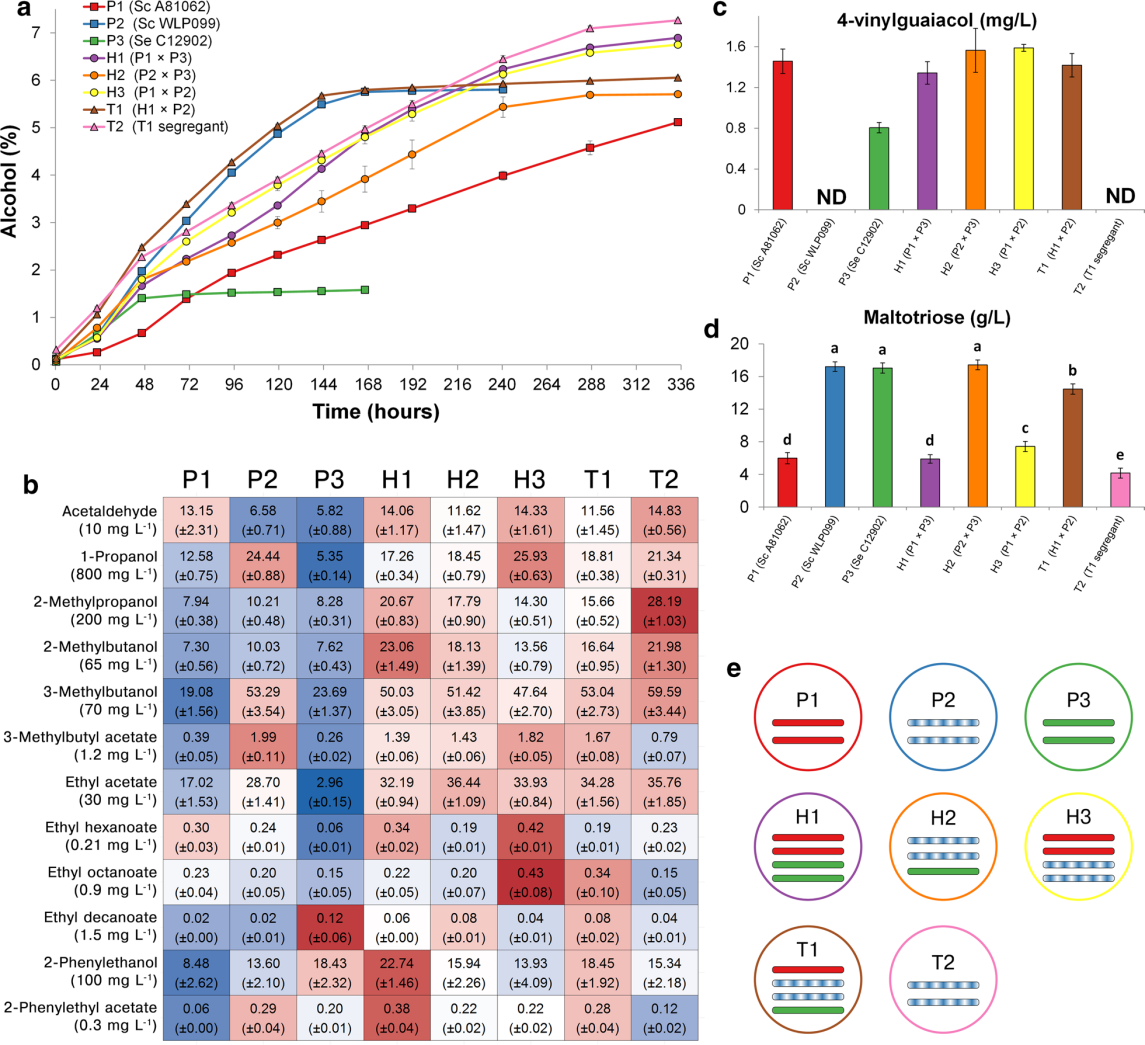
The **a** alcohol content (% ABV), **b** higher alcohol and ester concentrations (mg L^−1^), **c** 4-vinyl guaiacol concentration (mg L^−1^), and **d** maltotriose concentration (g L^−1^) in the beers fermented from the 15 °P wort with the 8 brewing strains. **a**, **c**, **d** Values are means from two independent fermentations and error bars where visible represent the standard deviation. Maltotriose concentrations with different superscript letters (*a*–*e*) differ significantly (*p* < 0.05). *ND* not detected. **b** The heat map was generated based on z-scores (*blue* and *red* indicate low and high values, respectively). The values in parentheses under the compound names represent the flavor threshold [65, 66]. Values are means from two independent fermentations (standard deviation in parentheses) and they have not been normalized to the ethanol concentration. **e** The *PAD1* alleles detected in the whole genome sequences of the 8 brewing strains. The alleles from the parent strains P1, P2 and P3 are depicted in *red*, *blue* and *green bars* respectively. A striped bar (P2) depicts a sequence containing single nucleotide polymorphisms (SNPs) possibly resulting in loss-of-function. The SNPs that were detected in the sequences of *PAD1* and *FDC1* are presented in Additional file 1: Table S3

To examine the performance of the 8 brewing strains in a brewery environment, fermentations were carried out in high-gravity 15 °P all-malt wort at 15 °C. These conditions were chosen to replicate those of industrial lager fermentations. There was considerable variation in fermentation performance between the eight strains, as is revealed by the wort alcohol content over time (Fig. 8a). Of the 8 strains, Hybrid T1 and *S. cerevisiae* P2 had the highest overall fermentation rates, but these slowed down considerably after reaching 5.8% (v/v) alcohol. Analysis of the sugar concentrations in the beer revealed that *S. cerevisiae* P2 was unable to ferment the maltotriose in the wort, while Hybrid T1 had only consumed approx. 15% of the initial maltotriose present in the wort (Fig. 8d). Of the 8 strains, Hybrid T2 reached the highest beer alcohol content [7.3% (v/v)], followed closely by Hybrids H1 and H3 (Fig. 8a). The maltotriose concentrations in the beers revealed that maltotriose had been efficiently used by these three hybrids (Fig. 8d). *S. cerevisiae* P1 had also used maltotriose efficiently, but it was only able to produce 5.1% (v/v) alcohol after 14 days of fermentation. *S. eubayanus* P3 on the other hand, was also unable to use maltotriose and the strain performed poorly in the wort reaching only 1.6% (v/v) alcohol.

The beers produced with the 8 brewing strains also varied considerably in concentrations of aroma-active compounds (Fig. 8b). The most ester-rich beers were produced with Hybrids H1, H3 and T1, and these contained as high or higher concentrations of most esters compared to either of the parent strains. Comparing the aroma profiles of the beers made with Hybrids T1 and T2, it is revealed that the meiotic segregant T2 produced lower concentrations of most esters, while its beer contained higher concentrations of most higher alcohols. This would suggest lower activities or expression of alcohol acetyl transferases in Hybrid T2. Of the 8 brewing strains, the POF− *S. cerevisiae* P2 and Hybrid T2 strains were the only ones that did not produce any detectable amounts of 4-vinyl guaiacol (detection limit 0.2 mg L^−1^), thus confirming their POF− phenotype (Fig. 8c). All other strains produced 4-vinyl guaiacol in concentrations above the flavour threshold of 0.3–0.5 mg L^−1^ [36]. Yeasts produce 4-vinyl guaiacol from ferulic acid with the aid of the *PAD1*- and *FDC1*-encoded enzymes. By comparing the sequences of these two genes in the 8 brewing strains, we found that Hybrid T2 only carried the *PAD1* allele that was derived from *S. cerevisiae* P2 (Fig. 8e; Additional file 1: Table S3). This particular allele, contained a possible loss-of-function SNP at position 638 (A>G, resulting in an amino acid substitution of aspartate to glycine). The other strains, including Hybrid T1, which Hybrid T2 was derived from, carried either or both of the functional *PAD1* alleles derived from *S. cerevisiae* P1 or *S. eubayanus* P3. While the alleles of *FDC1* in the parent strains contained different SNPs (Additional file 1: Table S3), none of them appeared to cause loss-of-function.

**Fig. 9:**
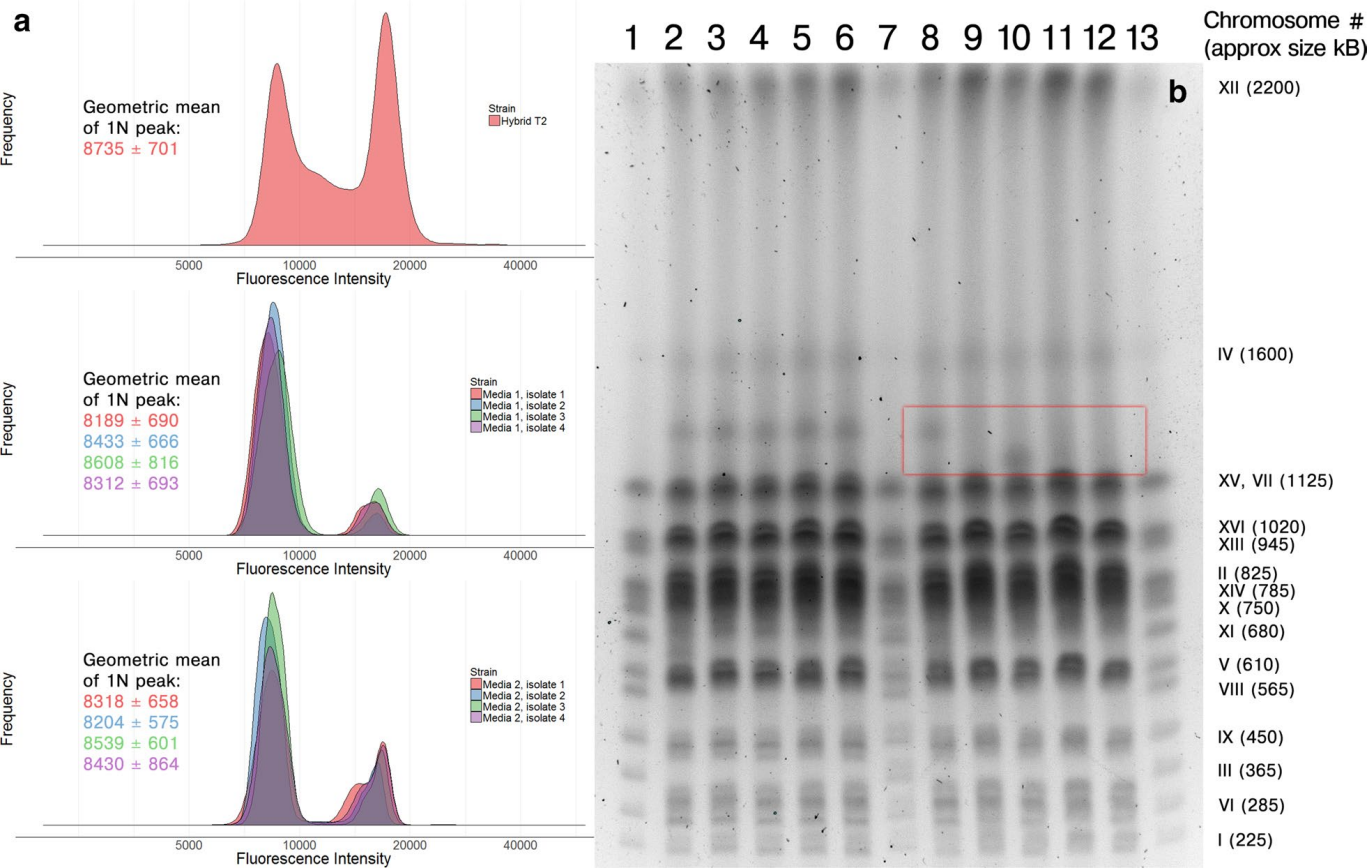
The karyotype stability of Hybrid T2 was assessed after 10 successive batch cultures. **a** The DNA content of Hybrid T2, the 4 isolates obtained after 10 cultures in Media 1, and the 4 isolates obtained after 10 cultures in Media 2 as determined by flow cytometry. The displayed values are the geometric mean and standard deviation of the 1 N peak. All 8 isolates showed a significant decrease in DNA content (*p* < 0.05 as determined by a two-sample Kolmogorov–Smirnov test). **b** PFGE separation of chromosomes from Hybrid T2 and 8 isolates obtained after 10 cultures in Media 1 and 2.* Lanes* 1, 7 and 13 chromosome marker strain YNN295,* lanes* 2 and 8 Hybrid T2,* lanes* 3–6 the 4 isolates obtained after 10 cultures in Media 1, and* lanes* 9–12 the 4 isolates obtained after 10 cultures in Media 2. The *red box* depicts an area where differences in chromosomes were observed in the isolates compared to Hybrid T2. Reference chromosomes are identified on the right: chromosomes VII and XV are not resolved; chromosome II travels immediately above chromosome XIV

During industrial beer fermentations, the yeast is commonly reused for multiple consecutive fermentations (typically up to 10 times depending on brewery), and it is therefore essential that the yeast genome maintains stable. In order to further evaluate the industrial applicability of Hybrid T2, its karyotype stability was assessed. After 10 successive batch cultures in two different growth media (Media 1 containing 1% yeast extract, 2% peptone, 1% maltose and 1% maltotriose; and Media 2 containing 1% yeast extract, 2% peptone, 2% maltose and 14% sorbitol), there was a significant decrease (*p* < 0.05) in DNA content for all eight 10-week isolates compared to the original strain (Fig. 9a). Pulsed-field gel electrophoresis (PFGE) further revealed that the 4 isolates obtained from Media 1 showed identical chromosome profiles as Hybrid T2 (Fig. 9b), suggesting that differences in DNA content could be a result of differences in chromosome copy number rather than complete loss of a chromosome. The 4 isolates obtained from Media 2 on the other hand, showed a loss of a whole or part of a chromosome (size between 1125 and 1600 kB) compared to Hybrid T2 (area highlighted with a red box in Fig. 9b). These results highlight the importance of stabilisation before any hybrids can be seen as viable candidates for industrial beer fermentations.

## Discussion

Interspecific hybridization has been shown to be a promising tool for increasing the diversity of lager yeasts available to the brewing industry. However, de novo lager yeast hybrids have so far inadvertently possessed a POF+ phenotype; a trait which they inherit from the *S. eubayanus* parent. In this study, we demonstrate the possibility of using an allotetraploid interspecific hybrid as an intermediate to create a POF− lager hybrid with DNA from three parent strains, and we use a set of brewing yeast hybrids to reveal that unsaturated fatty acids and ergosterol are associated with good fermentation performance at low temperatures and high ethanol concentrations.

Sporulation efficiency and spore viability tend to be poor in interspecies yeast hybrids, which limits the possibility of introducing variation to such a hybrid through meiotic recombination and chromosomal cross-over. The mechanisms contributing to hybrid sterility are not completely understood, but studies suggests that large sequence divergence, abnormal chromosome segregation and reciprocal gene loss contribute to it [9]. However, hybrid fertility can be recovered through a number of ways, one of which is genome doubling. Studies have revealed that doubling the genome content of sterile allodiploid hybrids result in allotetraploids capable of producing viable spores [6, 8]. Here, the allotetraploid interspecies hybrids H1 and T1 showed spore viabilities of 47 and 38%, respectively, and sporulation allowed us to not only cross Hybrid H1 with a third parent strain (P2), but also remove the POF+ phenotype from Hybrid T1. As was revealed from the genome sequences of Hybrids T1 and T2, chromosome losses may occur during spore formation (e.g. the *S. eubayanus*-derived chromosome XIII carrying *PAD1* and *FDC1*), suggesting that genetically diverse strains can be obtained. Hence, the use of fertile intermediates allows for the construction of complex interspecific hybrid strains, which can be screened and selected to contain desirable traits. While not investigated here, one may expect that meiotic segregants of interspecies hybrids vary considerably phenotypically as a result of orthologous gene segregation and the creation of unique biochemical pathways and regulatory mechanisms [37, 38]. We believe this would be particularly beneficial for generating novel and diverse lager yeast strains, as there currently exist limited genetic and phenotypic diversity between natural lager yeast hybrids [1, 39]. As the stability test of Hybrid T2 revealed that some genetic changes occurred after 10 successive batch cultures in relatively non-stressful media, it is vital that any hybrids are stabilised and phenotypically reassessed before they are viable candidates for industrial beer fermentations. Previous studies have shown that sufficient stabilisation can be achieved by growing the hybrids for 30–70 generations under fermentative conditions in high-sugar media [4, 40]. The inherent instability of interspecific yeast hybrids could also be exploited for further strain development through adaptive evolution, as was recently demonstrated for biofuel applications [41].

Phenolic off-flavours are undesirable in many beer styles, and in lager beer especially their presence is considered a flaw [36]. Hybridization and subsequent sporulation has been proposed as one technique of removing the POF+ phenotype from crossed *S. cerevisiae* strains [16]. Gallone et al. [13] have also shown that a POF− hybrid can be constructed when both parent strains carry *PAD1* or *FDC1* alleles containing loss-of-function mutations. *S. eubayanus* however, contains functional alleles of both *PAD1* and *FDC1,* so any hybrids made from it will initially be POF+ as well. Here we obtained a POF− interspecies hybrid through the use of a fertile tetraploid intermediate, and demonstrated that it didn’t produce 4-vinyl guaiacol in wort fermentations and only contained *PAD1* and *FDC1* alleles derived from the POF− *S. cerevisiae* P2 parent. No nonsense or frameshift mutations were detected in the *PAD1* and *FDC1* alleles of P2, but a SNP at position 638 (A>G) of *PAD1* results in an Asp213Gly amino acid substitution, possibly affecting the functionality of the enzyme. This same SNP (*PAD1*: 638 A>G) was present in the POF− K1V-1116 strain that was studied by Mukai et al. [14] and the POF− strains Beer024, Beer033, Beer088, Wine001, Wine009 and Spirits002 that were studied by Gallone et al. [13]. However, it may be possible that this SNP is not the cause for the POF− phenotype in P2 and T2, as well as the POF− strains from the previously mentioned studies, and rather another unknown mechanism is responsible.

Among the defining characteristics of lager yeast is their ability to grow and ferment at low temperatures and high ethanol concentrations. Unlike the POF+ phenotype, the mechanisms that contribute to cold and ethanol tolerance in brewing yeast are more complex and not fully understood. Differences in the lipid composition of the plasma membrane have however been shown to play a vital role in determining both temperature and ethanol tolerance [25–27, 33]. Here, we observed variations in cellular lipid composition both between yeast strains and between environmental conditions. When temperatures were lowered or ethanol content was increased, the degree of unsaturation tended to increase. Similar conclusions have also been reached in recent studies in wine-making conditions [25, 26, 32]. It is assumed that these changes in response to a lowered temperature or an increase in ethanol concentration are vital for maintaining membrane fluidity and functionality [25, 33, 42]. The PLS models that were constructed between the lipid dataset and fermentation parameters at the different conditions revealed that yeast strains which performed well at low temperatures and/or high ethanol concentrations were associated with increased concentrations of phospholipids and triacylglycerides containing unsaturated fatty acids, such as palmitoleic acid (C16:1) and oleic acid (C18:1). Ergosterol was also shown to be positively correlated with ethanol tolerance, particularly at 20 °C. Studies have revealed that ergosterol plays an important role in maintaining membrane rigidity and protecting against ethanol toxicity when ethanol concentrations are increased [30, 31]. Results from microplate cultivations of *ole1*∆ and *erg4*∆ knockout strains supplemented with oleic acid and ethanol were in agreement with these observations. Redón et al. [35] also observed that wine fermentations at low temperatures proceeded faster when unsaturated fatty acids such as palmitoleic and linolenic acid were supplemented to the must. Despite the PLS models revealing that lipid composition does seem to influence cold and ethanol tolerance in brewing yeast, we feel that the lipidomics data generated here did not reveal any obvious patterns between the two. It is thus plausible that a combination of other factors, e.g. protein translation and folding efficiencies, mRNA stabilities and the product activity and expression of central metabolic genes [18–24], also contribute to determining cold and ethanol tolerance, and suggest that this topic should be addressed in future studies.

## Conclusions

Recent studies have revealed that the creation of de novo lager yeast hybrids has the potential to considerably increase the genetic and phenotypic diversity of lager yeast strains available to the brewing industry. During interspecific hybridization, one is typically limited to combining traits from two different strains. Here we demonstrated the possibility of constructing complex yeast hybrids, through the use of a fertile allotetraploid intermediate, that possess traits that are relevant to industrial lager beer fermentation and that are derived from several parent strains. Yeast lipid composition was also shown to have a central role in determining ethanol and cold tolerance in brewing strains. The presence of unsaturated fatty acids and ergosterol in particular, were shown to benefit growth in the presence of ethanol and at lower temperatures.

## Methods

### Yeast strains and hybrid generation

The yeast strains used in the study are listed in Table 1. The interspecific yeast hybrids H1 (P1 × P2) and H2 (P2 × P3) and intraspecific yeast hybrid H3 (P1 × P2) were generated using rare mating according to the method described in Krogerus et al. [3]. Prior to mating, a uracil auxotroph (*ura*-) of P1 was selected on 5-fluoroorotic acid (5-FOA) agar [43], a respiratory-deficient mutant (*rho*-) of P2 was selected on YPDG agar containing 3% glycerol and 0.1% glucose, and a lysine auxotroph (*lys*-) of P3 was selected on α-aminoadipic (α-AA) agar [44] to allow for the selection of hybrids on minimal selection agar medium (0.67% Yeast Nitrogen Base without amino acids, 3% glycerol, 3% ethanol and 2% agar). The interspecific hybrid H1 was further mated with P2 (*rho*-) to form the interspecific triple hybrid T1 [(P1 × P3) × P2]. Prior to mating, a lysine auxotroph (*lys*-) of H1 was first selected as described above, after which ascospores of it were generated on 1% potassium acetate agar. Spore viability was calculated based on the amount of colonies formed from the dissection of up to 20 tetrads. Ascus walls were digested with Zymolyase 100T, after which spores of H1 (*lys*-) were mixed with P2 (*rho*-). Hybrid T1 was selected on minimal selection agar. Hybrid T2, a meiotic segregant of T1, was created by generating ascospores of Hybrid T1 on 1% potassium acetate agar and then dissecting the spores on YPD agar. All spore dissections were carried out using the Singer MSM 400 dissecting microscope (Singer Instruments, UK). The viable spore clones were then screened for the POF phenotype in a small-scale assay. 1 ml of YPM supplemented with 100 mg L^−1^ of *trans*-ferulic acid was inoculated with a colony of the spore clones, and they were allowed to incubate for 48 h at 25 °C. The POF phenotype was determined sensorially by examining for the presence (POF+) or absence (POF−) of the distinct clove-like aroma of 4-vinyl guaiacol. Hybrid T2 was a spore clone of T1 which did not produce 4-vinyl guaiacol in this assay. An overview of these brewing strains and their creation is presented in Fig. 1.

The hybrid status of isolates was confirmed by PCR as described in Krogerus et al. [2]. Briefly, the rDNA ITS region was amplified using the primers ITS1 (5′-TCCGTAGGTGAACCTGCGG-3′) and ITS4 (5′-TCCTCCGCTTATTGATATGC-3′) and amplicons were digested using the HaeIII restriction enzyme (New England BioLabs, USA) as described previously [45]. Amplification of the *S. eubayanus*-specific *FSY1* gene (amplicon size 228 bp) and the *S. cerevisiae*-specific *MEX67* gene (amplicon size 150 bp) was also performed using the primers SeubF3 (5′-GTCCCTGTACCAATTTAATATTGCGC-3′), SeubR2 (5′-TTTCACATCTCTTAGTCTTTTCC-AGACG-3′), ScerF2 (5′-GCGCTTTACATTCAGATCCCGAG-3′), and ScerR2 (5′-TAAGTTGGTTGTCAGCAAGATTG-3′) as described by Muir et al. [46] and Pengelly and Wheals [47]. Additionally, the hybrid statuses of the intraspecific hybrid H3 and the triple hybrids T1 and T2 were confirmed by amplifying interdelta sequences using the delta12 (5′-TCAACAATGGAATCCCAAC-3′) and delta21 (5′-CATCTTAACACCGTATATGA-3′) primers as described by Legras and Karst [48].

Flow cytometry was also performed on the brewing yeast strains to estimate ploidy essentially as described by Haase and Reed [49]. Cells were grown overnight in YPD medium (1% yeast extract, 2% peptone, 2% glucose), and approximately 1 × 10^7^ cells were washed with 1 mL of 50 mM citrate buffer. Cells were then fixed with cold 70% ethanol, and incubated at room temperature for 1 h. Cells were then washed with 50 mM citrate buffer (pH 7.2), resuspended in 50 mM citrate buffer containing 0.25 mg mL^−1^ RNAse A and incubated overnight at 37 °C. 1 mg mL^−1^ of Proteinase K was then added, and cells were incubated for 1 h at 50 °C. Cells were then stained with SYTOX Green (2 μM; Life Technologies, USA), and their DNA content was determined using a FACSAria IIu cytometer (Becton–Dickinson, USA). DNA contents were estimated by comparing fluorescence intensities with those of *S. cerevisiae* haploid (CEN.PK113-1A) and diploid (CEN.PK) reference strains. Measurements were performed on duplicate independent yeast cultures, and 100,000 events were collected per sample during flow cytometry.

### Genome sequencing

Whole genome sequences of the brewing strains P1 and P3 have been published previously [3, 50]. The other 6 brewing strains were sequenced by Biomedicum Genomics (Helsinki, Finland). In brief, an Illumina NexteraXT pair-end 150 bp library was prepared for each hybrid and sequencing was carried out with a NextSeq500 instrument. Pair-end reads from the NextSeq500 sequencing were quality-analysed with FastQC [51] and trimmed and filtered with Skewer [52]. Alignment, re-alignment and variant analysis was carried out using SpeedSeq [53] and its FreeBayes SNP prediction and CNVnator copy number variation prediction modules [54, 55]. Reads of *S. cerevisiae* P2 were aligned to that of *S. cerevisiae* S288c [56], while reads of hybrid strains were aligned to concatenated reference sequences of strains P1 and P3 [3, 50] as described previously [3]. SNPs predicted by FreeBayes with less than five left and right aligning reads were discarded. Prior to SpeedSeq variant analysis, alignments were filtered to a minimum MAPQ of 50 with SAMtools [57]. Quality of alignments was assessed with QualiMap [58]. The median coverage over 10,000 bp windows was calculated with BEDTools [59] and visualized with R (http://www.r-project.org/). The coverage of *S. cerevisiae* P2 and the 5 hybrid strains (H1-H3 and T1-T2) across the *S. cerevisiae* and *S. eubayanus* reference genomes are displayed in Additional file 1: Figure S3.

### Fermentation assay

The fermentation kinetics and lipid compositions of the 8 brewing strains at two different temperatures (10 and 20 °C) and two different initial ethanol concentrations [0 and 8% (v/v)] were assayed in 100 mL shake-flask fermentations. The strains were grown in media containing 1% yeast extract, 2% peptone, 8% maltose, and up to 8% ethanol. Prior to the assay, the strains were pre-cultivated in media containing 1% yeast extract, 2% peptone and 4% maltose for 24 h at 20 °C. The OD600 of the pre-cultivations was measured, and the growth assays were started by inoculating 100 mL of media to a starting OD600 of 0.01 in triplicate flasks. Flasks were capped and then incubated at either 10 or 20 °C with light agitation (80 RPM) for up to 33 days. Fermentation was monitored (up to twice daily) by drawing 100 μL samples and measuring the refractive index (°brix) with a Quick-Brix 90 digital refractometer (Mettler-Toledo AG, Switzerland). Samples were drawn for lipid analysis at the end of the exponential fermentation phase. 15 mL samples of fermentation media were centrifuged for 5 min at 9000×*g*, after which the yeast pellet was washed twice in 15 mL of ice-cold deionized water. The washed yeast pellet was then transferred to a cryotube, and flash-frozen in liquid nitrogen. The samples were stored at −80 °C prior to lipid analysis. After fermentations were complete, the biomass concentration determined by drawing and centrifuging 30 mL samples of the fermentation media (10 min at 9000×*g*), washing the yeast pellets gained from centrifugation twice with 25 mL deionized H_2_O and then suspending the washed yeast in a total of 6 mL deionized H_2_O. The suspension was then transferred to a pre-weighed porcelain crucible, and was dried overnight at 105 °C and allowed to cool in a desiccator before the change of mass was measured. Fermentation curves for the fermentations were modelled based on the decrease in °brix over time using the ‘grofit’-package for R [60]. The fermentation parameters were determined using the logistic model in ‘grofit’.

### Lipid analysis

Prior to lipid extraction, the frozen cell samples were freeze-dried at −55 °C overnight (Martin Christ Alpha 1-4 LDplus, Germany). For fatty acid (free and bound) and sterol analysis, 10 mg of freeze-dried sample was rehydrated into 200 µL of 0.9% sodium chloride solution and spiked with heptadecanoic acid (FFA 17:0; 5.36 µg) and glyceryl triheptadecanoate [TG(17:0/17:0/17:0); 21.11 µg]. Lipids were extracted with chloroform:methanol (2:1 v/v; 800 µL) by homogenizing the samples with grinding balls in a Retsch mixer mill MM400 homogenizer (Retsch GmbH, Haan, Germany) at 20 Hz for 2 min. After 30 min standing at room temperature, the samples were centrifuged at 10,620×*g* for 5 min and the lower layer was separated into glass tubes and evaporated to dryness under nitrogen flow.

The evaporation residues from lipid extractions were dissolved into petroleum ether (b.p. 40–60 °C; 700 µL). Fatty acids were transesterified with sodium methoxide (NaOMe; 0.5 M; 250 µL) in dry methanol by boiling at 45 °C for 5 min. The mixture was acidified with 15% sodium hydrogen sulphate (NaHSO_4_; 500 µL). The petroleum ether phase containing the fatty acid methyl esters (FAME) as well as free fatty acids (FFA) was collected into an Eppendorf tube and centrifuged (10,620×*g*; 5 min). Half of the petroleum ether layer was separated into a GC vial and evaporated, after which the residue was dissolved into hexane (100 µL) and taken into a vial insert. FAMEs were analysed on an Agilent 7890A GC combined with an Agilent 5975C mass selective detector controlled by MSD ChemStation software (Agilent Technologies Inc., Santa Clara, CA, USA). The column was an Agilent FFAP silica capillary column (25 m × 0.2 mm × 0.3 µm). Helium was used as carrier gas and the samples were injected in splitless mode. The oven temperature programme was from 70 °C (2 min) to 240 °C at a rate of 15 °C min^−1^, total run time was 39 min. The temperatures of the injector and MS source were 260 and 230 °C, respectively. The samples (1 µL) were injected by a Gerstel MPS injection system (Gerstel GmbH & Co. KG, Mülheim an der Ruhr, Germany) and the data were collected in EI mode (70 eV) at a mass range of m/z 40–600.

The other half of the petroleum ether layer was transferred to a GC vial and evaporated into dryness for the determination of free fatty acid and sterols. The residue was dissolved into 50 µL of DCM and derivatized with *N*-Methyl-*N*-(trimethylsilyl)trifluoroacetamide (MSTFA; 40 µL) and trimethylchlorosilane (TMCS; 10 µL) by incubating at 80 °C for 20 min. The samples (1 µL aliquots) were injected in splitless mode at 300 °C, and analysed on an Agilent DB-5MS column (30 m × 0.2 mm × 0.25 µm). The oven temperature programme was from 50 °C (1.5 min) to 325 °C at a rate of 10 °C min^−1^, total run time was 49 min.

For lipidomics analyses, approx. 5 mg of freeze-dried cell samples were rehydrated in 50 µL of 0.9% sodium chloride in Eppendorf tubes, mixed with 400 µL chloroform:methanol (2:1) and spiked with 10 µL of an internal standard mixture 1 [IS1; containing LysoPC(17:0), MG(17:0), DG(17:0/17:0), TG(17:0/17:0/17:0), PG(17:0/17:0), Cer(d18:1/17:0), PC(17:0/17:0), PE(17:0/17:0), CE(19:0), CA(14:0), C12(β)-d-GluCer and C8-l-threo-LacCer] (Avanti Polar Lipids, Alabaster, AL, USA; Larodan Fine Chemicals AB, Malmö, Sweden; Nu-Chek Prep, Inc., Elysian, MN, USA) at concentration levels of 0.4–3.2 µg/sample. The samples were homogenized with grinding balls in a Retsch mixer mill MM400 homogenizer at 25 Hz for 2 min and after 30 min standing were centrifuged at 10,620×*g* for 3 min. Before UPLC-MS analysis, a 20 µL aliquot of a labelled lipid standard mixture [IS2; containing LysoPC(16:0-D_3_), PC(16:0/16:0-D_6_) and TG(16:0/16:0/16:0-^13^C3)] (Avanti Polar Lipids, Alabaster, AL, USA) was added into the separated lipid extracts.

Lipid extracts were analyzed on a Waters Q-TOF Premier mass spectrometer combined with an Acquity Ultra Performance LC™ (UPLC) under the control of MassLynx software (v 4.1; Waters Inc., Milford, MA, USA). The column (at 50 °C) was an Acquity UPLC™ BEH C18 (2.1 × 100 mm with 1.7 μm particles). The solvent system included (A) ultrapure water (1% 1 M NH_4_Ac, 0.1% HCOOH) and (B) LC/MS grade acetonitrile/isopropanol (1:1, 1% 1 M NH_4_Ac, 0.1% HCOOH). In ESI− mode, the same solvent system but without acid was used. The gradient started from 65% A/35% B, reached 80% B in 2 min, 100% B in 7 min and remained there for the next 7 min. There was a 5 min re-equilibration step before the next run. The flow rate was 0.400 mL min^−1^ and the injected amount 1.0 μL (Acquity Sample Organizer at 10 °C). The ESI source was at 120 °C and the capillary voltage 3.0 and 2.5 kV in positive and negative mode, respectively. N_2_ was used as desolvation gas (800 L h^−1^) at 270 °C.

The data were collected at a mass range of m/z 300–1200 with a scan duration of 0.2 s. Reserpine was used as the lock spray reference compound in ESI+ mode and leucine enkephaline in ESI− mode. Data processing was carried out with MZmine software (version 2.20) [61] enabling peak integration and alignment of the peaks. An internal spectral MS/MS library was used for identification of the compounds.

Quantification of lipid subspecies was based on peak heights of internal standards. All monoacyl lipids except cholesterol esters, such as monoacylglycerols and monoacylglycerophospholipids, were normalized with LysoPC(17:0), all diacyl lipids except ethanolamine phospholipids were normalized with PC(17:0/17:0), all ceramides with Cer(d18:1/17:0), all diacyl ethanolamine phospholipids with PE(17:0/17:0), and TGs and sterylesters were normalized with TG(17:0/17:0/17:0) and CE(19:0), respectively. Other (unidentified) molecular species were normalized with LysoPC(17:0) for retention time <300 s, PC(17:0/17:0) for retention time between 300 and 410 s, and TG(17:0/17:0/17:0) for higher retention times.

### Microplate cultivations

To assess the roles of oleic acid (C18:1) on cold and ethanol tolerance in yeast, microcultures were carried out in media containing various concentrations of supplemented oleic acid (0 or 0.8 mM) and ethanol (1, 5 or 10% v/v). This concentration of oleic acid was chosen based on values found previously in literature [35]. The microcultures were carried out in 100-well honeycomb microtiter plates at 15 and 20 °C (with continuous shaking), and their growth dynamics were monitored with a Bioscreen C MBR incubator and plate reader (Oy Growth Curves Ab, Finland). The wells of the microtiter plates were filled with 300 µL of YPDt medium (1% yeast extract, 2% peptone, 2% glucose, and 1% Tergitol NP-40) supplemented with oleic acid (0 or 0.8 mM) and ethanol (1, 5 or 10% v/v). Oleic acid was added to the media from a 100 × stock solution (80 mM oleic acid) prepared in 50% ethanol and 35% Tergitol NP-40. Precultures of the laboratory strains WT, *ole1*∆, and *erg4*∆ (Table 1) were started in 10 mL YPD medium (1% yeast extract, 2% peptone, and 2% glucose) and incubated at 25 °C with shaking at 120 rpm. The cultures were centrifuged and the yeast pellets were washed once with sterile deionized water. The yeast was then resuspended in YPDt medium to an OD600 value of 10. The microcultures were started by inoculating the microtiter plates with 3 µL of cell suspension per well (for an initial OD600 value of 0.1) and placing the plates in the Bioscreen C MBR. The optical density of the microcultures at 600 nm was automatically read every 30 min. Four replicates were performed for each strain in each medium. Growth curves for the microcultures were modelled based on the OD600 values over time using the ‘grofit’-package for R [60].

### Fermentation and analysis

The set of eight brewing strains were characterized in fermentations performed in a 15 °Plato high gravity wort at 15 °C. Yeast was propagated essentially as described previously [3], with the use of a ‘Generation 0’ fermentation prior to the actual experimental fermentations. The experimental fermentations were carried out in duplicate, in 2-L cylindroconical stainless steel fermenting vessels, containing 1.5 L of wort medium. The 15 °Plato wort (69 g maltose, 17.4 g maltotriose, 15.1 g glucose, and 5.0 g fructose per litre) was produced at the VTT Pilot Brewery from barley malt. Yeast was inoculated at a rate of 15  ×  10^6^ viable cells mL^−1^. The wort was oxygenated to 15 mg L^−1^ prior to pitching (Oxygen Indicator Model 26073 and Sensor 21158, Orbisphere Laboratories, Switzerland). The fermentations were carried out at 15 °C until an apparent attenuation of 80% (corresponding to approx 7% ABV) was reached, until no change in residual extract was observed for 24 h, or for a maximum of 14 days.

Wort samples were drawn regularly from the fermentation vessels aseptically, and placed directly on ice, after which the yeast was separated from the fermenting wort by centrifugation (9000×*g*, 10 min, 1 °C). Samples for yeast-derived flavour compounds, fermentable sugars and 4-vinyl guaiacol analysis were drawn from the beer when fermentations were ended. Yeast viability was measured from the yeast that was collected at the end of the fermentations using a Nucleocounter^®^ YC-100™ (ChemoMetec, Denmark).

The alcohol level (% v/v) and pH of samples was determined from the centrifuged and degassed fermentation samples using an Anton Paar Density Meter DMA 5000 M with Alcolyzer Beer ME and pH ME modules (Anton Paar GmbH, Austria). The yeast dry mass content of the samples (i.e. yeast in suspension) was determined by washing the yeast pellets gained from centrifugation twice with 25 mL deionized H_2_O and then suspending the washed yeast in a total of 6 mL deionized H_2_O. The suspension was then transferred to a pre-weighed porcelain crucible, and was dried overnight at 105 °C and allowed to cool in a desiccator before the change of mass was measured. The measured pH values and suspended dry mass are presented in Additional file 1: Figure S4.

Concentrations of fermentable sugars (glucose, fructose, maltose and maltotriose) were measured by HPLC using a Waters 2695 Separation Module and Waters System Interphase Module liquid chromatograph coupled with a Waters 2414 differential refractometer (Waters Co., Milford, MA, USA). An Aminex HPX-87H Organic Acid Analysis Column (300 × 7.8 mm, Bio-Rad, USA) was equilibrated with 5 mM H_2_SO_4_ (Titrisol, Merck, Germany) in water at 55 °C and samples were eluted with 5 mM H_2_SO_4_ in water at a 0.3 mL min^−1^ flow rate.

Yeast-derived flavour compounds were determined by headspace gas chromatography with flame ionization detector (HS-GC-FID) analysis. 4 mL samples were filtered (0.45 µm), incubated at 60 °C for 30 min and then 1 mL of gas phase was injected (split mode; 225 °C; split flow of 30 mL min^−1^) into a gas chromatograph equipped with an FID detector and headspace autosampler (Agilent 7890 Series; Palo Alto, CA, USA). Analytes were separated on a HP-5 capillary column (50 m × 320 µm × 1.05 µm column, Agilent, USA). The carrier gas was helium (constant flow of 1.4 mL min^−1^). The temperature program was 50 °C for 3 min, 10 °C min^−1^ to 100 °C, 5 °C min^−1^ to 140 °C, 15 °C min^−1^ to 260 °C and then isothermal for 1 min. Compounds were identified by comparison with authentic standards and were quantified using standard curves. 1-Butanol was used as internal standard.

4-Vinyl guaiacol was analyzed using HPLC-PAD based on methods described by Coghe et al. [62] and McMurrough et al. [63]. The chromatography was carried out using a Waters Alliance HPLC system consisting of a Waters e2695 Separations Module equipped with a XTerra^®^ MS C18 column (5 µm, 4.6 × 150 mm) and a Waters 2996 Photodiode Array Detector. The mobile phase consisted of H_2_O/CH_3_OH/H_3_PO_4_ (64:35:1, v/v) and flow rate was 0.5 mL min^−1^. The diode array detector was used at 190–400 nm. 4-Vinyl guaiacol was quantified at 260 nm using standard curves of the pure compound (0.3–30 mg L^−1^).

### Stability of Hybrid T2

The karyotype stability of Hybrid T2 was evaluated after 10 successive batch cultures (corresponding to approximately 65 cell generations) in two different media. Media 1 consisted of 1% yeast extract, 2% peptone, 1% maltose and 1% maltotriose, while Media 2 consisted of 1% yeast extract, 2% peptone, 2% maltose and 14% sorbitol. Media 2 was used to mimic the osmotic stress occurring during high-gravity wort fermentations. Hybrid T2 was first grown overnight in YPM at 25 °C. This culture was used to inoculate 1 mL of Media 1 or Media 2 in duplicate to a starting OD600 of 0.1. The cultures were allowed to grow for 7 days at 18 °C, after which they were used to inoculate a fresh 1 mL aliquot of Media 1 or Media 2 to a starting OD600 of 0.1. This was repeated for a total of 10 successive cultures (10 weeks). After this, 20 μL aliquots of the cultures were spread out on YPM agar, and a total of 8 colonies were randomly selected and isolated for further analysis (2 isolates per duplicate per media).

The karyotype stability of the 10-week isolates was assessed by determining their DNA content by flow cytometry as described above, and their karyotypes by pulsed-field gel electrophoresis (PFGE). PFGE was carried out essentially as described previously [2]. Sample plugs were prepared with the CHEF Genomic DNA Plug Kit for Yeast (Bio-Rad) according to the manufacturer’s instructions with minor modifications. Instead of lyticase treatment, the plugs were treated with 0.1 mg mL^−1^ Zymolyase 100T in buffer containing 1 mM dithiothreitol. The sample plugs were loaded into the wells of a 1.0% pulse field certified agarose (Bio-Rad) gel. PFGE was performed at 14 °C in 0.5 × TBE buffer [89 mM Tris, 89 mM boric acid, 2 mM EDTA (pH 8)]. A CHEF Mapper XA pulsed field electrophoresis system (Bio-Rad) was used with the following settings: 6 V cm^−1^ in a 120° angle, pulse length increasing linearly from 26 to 228 s, and total running time of 40 h. A commercial chromosome marker preparation from *S. cerevisiae* strain YNN295 (Bio-Rad) was used for molecular mass calibration. After electrophoresis, the gels were stained with ethidium bromide and scanned with Gel Doc XR+ imaging system (Bio-Rad).

### Data analysis

Data and statistical analyses were performed with R (http://www.r-project.org/). The distributions of the lipidomic data were estimated by histograms and the Shapiro–Wilk test, and the lipidomic data was consequently log10-transformed to correct for skewed distributions. The change in lipid composition compared to the control cultivation at 20 °C and 0% supplemented EtOH was tested by Student’s t test (two-tailed, unpaired, and unequal variances). To control for multiple testing, the *p* values were further adjusted for Benjamini–Hochberg false discovery rate (FDR). Strain-specific differences in fatty acid, squalene and ergosterol concentrations were tested with one-way ANOVA with Tukey’s post hoc test. Multivariate analysis was performed with Partial Least Squares (PLS) and PLS-Discriminant Analysis (PLS-DA) using the ‘ropls’ package in R [64]. PLS-DA was initially performed on the lipid data of all samples in order to determine whether the lipid compositions of the yeast in low temperatures or high alcohol levels could be distinguished from those at control conditions (20 °C, 0% supplemented EtOH). PLS models were constructed from the fermentation and lipid data obtained at the different temperatures and supplemented ethanol levels in order to elucidate which lipid species contributed positively and negatively to fermentation performance at those conditions. The *Y* response variable of the models was the maximum fermentation level divided by the lag time (*A* and λ from Table 2, respectively), while the *X* predictor variables were the combined dataset of the compositions of fatty acids, squalene and ergosterol obtained from GC/MS analysis and the compositions of the 60 lipid species obtained from UPLC/MS lipidomics analysis. PLS(-DA) models were cross-validated (*Q* ^2^ > 0.5 was considered significant [34]), and the significance of the *Q* ^2^ value was tested with 200 random permutations of the *X* dataset.

## Additional files

**Additional file 1: Table S1.** The relative concentrations (% of total lipid content) of fatty acids, squalene and ergosterol, unsaturated to saturated fatty acid ratio, and average fatty acid chain length in the lipids extracted from cells in late exponential phase during the growth assays. **Table S2.** Modelled (*A*, μ, λ) growth parameters of the microplate cultivations performed with the three laboratory strains grown in media supplemented with 0.8 mM oleic acid and various concentrations of ethanol (growth curves are presented in Fig. 7 in the main article). **Table S3.** The alleles of the *PAD1* and *FDC1* genes (responsible for the ‘phenolic off-flavour’-phenotype) that were detected in the 8 brewing strains based on single nucleotide polymorphisms. **Figure S1.** Confirmation of hybridization by (**A**) interdelta PCR, (**B**) rDNA ITS PCR and RFLP, and (**C**) amplification of *FSY1* and *MEX67* genes using species-specific primers. **Figure S2.** DNA content of the (**A**) *S. cerevisiae* haploid (CEN.PK113-1A) and diploid (CEN.PK) reference strains, (**B**) P1-P3 parent strains (all diploid), (**C**) H1-H3 hybrid strains (allotetraploid, allotriploid and allotetraploid, respectively), and (**D**) T1-T2 hybrids strains (allotetraploid and allodiploid, respectively) by flow cytometry. **Figure S3.** The sequencing coverage (median in 10 kbp windows) over the *S. cerevisiae*- (black and red) and *S. eubayanus*-derived (black and blue) chromosomes of parent and hybrid strains (**A**) P2 (265x), (**B**) H1 (87x), (**C**) H2 (304x), (**D**) H3 (275x), (**E**) T1 (295x), and (**F**) T2 (317x). **Figure S4** The (**A**) suspended yeast dry mass (g L^−1^) and (**B**) pH in the beers fermented from the 15 °P wort with the 8 brewing strains.

**Additional file 2.** The masses (m/z), retention times (s), and identifications of 60 lipid subspecies in the 8 brewing strains at the 4 different growth conditions. The values represent relative amounts (%) of the 60 identified compounds out of the total 488 obtained in lipidomics analyses. The identified lipid species constitute an average of 43.5% of the total amount of lipids.

## Abbreviations

CA: cardiolipin
CE: cholesterol ester
Cer: ceramide
DG: diacylglyceride
FA: fatty acid
GluCer: glucosyl ceramide
LacCer: lactosyl ceramide
MG: monacylglyceride
PA: phosphatidic acid
PC: phosphatidylcholine
PE: phosphatidylethanolamine
PG: phosphatidylglycerol
PI: phosphatidylinositol
PLS: partial least squares
PLS-DA: partial least squares discriminant analysis
POF: phenolic off-flavour
PS: phosphatidylserine
SFA: saturated fatty acid
SNP: single nucleotide polymorphism
TG: triacylglyceride
UFA: unsaturated fatty acid
ZymoE: zymosterol ester
