# Supplementary material for: Inheritance of brewing-relevant phenotypes in constructed Saccharomyces cerevisiae × Saccharomyces eubayanus hybrids
Source: Microb Cell Fact. 2017 Apr 21;16:66. doi: 10.1186/s12934-017-0679-8 (PMC5399851; doi:10.1186/s12934-017-0679-8)
Supplement: Supplementary file 1 — Additional file 1: Table S1. The relative concentrations (% of total lipid content) of fatty acids, squalene and ergosterol, unsaturated to saturated fatty acid ratio, and average fatty acid chain length in the lipids extracted from cells in late exponential phase during the growth assays. Table S2. Modelled (A, μ, λ) growth parameters of the microplate cultivations performed with the three laboratory strains grown in media supplemented with 0.8 mM oleic acid and various concentrations of ethanol (growth curves are presented in Fig. 7 in the main article). Table S3. The alleles of the PAD1 and FDC1 genes (responsible for the ‘phenolic off-flavour’-phenotype) that were detected in the 8 brewing strains based on single nucleotide polymorphisms. Figure S1. Confirmation of hybridization by (A) interdelta PCR, (B) rDNA ITS PCR and RFLP, and (C) amplification of FSY1 and MEX67 genes using species-specific primers. Figure S2. DNA content of the (A) S. cerevisiae haploid (CEN.PK113-1A) and diploid (CEN.PK) reference strains, (B) P1-P3 parent strains (all diploid), (C) H1-H3 hybrid strains (allotetraploid, allotriploid and allotetraploid, respectively), and (D) T1-T2 hybrids strains (allotetraploid and allodiploid, respectively) by flow cytometry. Figure S3. The sequencing coverage (median in 10 kbp windows) over the S. cerevisiae- (black and red) and S. eubayanus-derived (black and blue) chromosomes of parent and hybrid strains (A) P2 (265x), (B) H1 (87x), (C) H2 (304x), (D) H3 (275x), (E) T1 (295x), and (F) T2 (317x). Figure S4 The (A) suspended yeast dry mass (g L−1) and (B) pH in the beers fermented from the 15 °P wort with the 8 brewing strains. [file 12934_2017_679_MOESM1_ESM.pdf]

Additional file 1

Inheritance of brewing-relevant phenotypes in constructed Saccharomyces cerevisiae × Saccharomyces eubayanus hybrids

Microbial Cell Factories

Kristoffer Krogerus[1,2*], Tuulikki Seppänen-Laakso[1], Sandra Castillo[1], Brian Gibson[1]

kristoffer.krogerus@aalto.fi,

tuulikki.seppanen-laakso@vtt.fi,

sandra.castillo@vtt.fi,

brian.gibson@vtt.fi

[1] VTT Technical Research Centre of Finland,

Tietotie 2, P.O. Box 1000,

FI-02044 VTT, Espoo,

Finland

[2] Department of Biotechnology and Chemical Technology,

Aalto University, School of Chemical Technology,

Kemistintie 1, Aalto, P.O. Box 16100,

FI-00076 Espoo,

Finland

* Corresponding author

Table S1 – The relative concentrations (% of total lipid content) of fatty acids, squalene and ergosterol, unsaturated (UFA) to saturated (SFA) fatty acid ratio, and average fatty acid (FA) chain length in the lipids extracted from cells in late exponential phase during the growth assays. Values are means from three independent cultivations (standard deviation in parentheses). Values in the same group (i.e. the same temperature and ethanol concentration) in the same column with different superscript letters (a–g) differ significantly ($p < 0.05$ as determined with one-way ANOVA with Tukey's post-hoc test). NA: not available.

Strain and condition		C14:0	C16:0	C16:1	C18:0	C18:1	Squalene	Ergosterol	UFA/SFA ratio	Average FA chain length
10 °C, 0% EtOH	P1	0.54 (±0.04)^{c}	6.64 (±0.18)^{d}	41.93 (±0.82)^{ef}	7.37 (±0.28)^{ab}	43.52 (±0.83)^{a}	0.12 (±0.03)^{d}	6.84 (±0.36)^{a}	5.88 (±0.20)^{a}	17.01 (±0.02)^{a}
	P2	0.62 (±0.08)^{c}	8.86 (±0.62)^{c}	39.15 (±1.12)^{g}	8.69 (±0.77)^{a}	42.68 (±1.49)^{ab}	0.30 (±0.12)^{d}	4.05 (±1.05)^{b}	4.53 (±0.46)^{bc}	17.02 (±0.03)^{a}
	P3	0.33 (±0.04)^{d}	9.22 (±0.51)^{c}	62.25 (±0.60)^{a}	3.99 (±0.73)^{d}	24.20 (±1.37)^{d}	1.30 (±0.28)^{bc}	2.34 (±0.59)^{c}	6.40 (±0.52)^{a}	16.56 (±0.01)^{d}
	H1 (P1 × P3)	0.62 (±0.03)^{c}	9.18 (±0.51)^{c}	52.55 (±1.25)^{b}	4.87 (±0.28)^{cd}	32.79 (±0.76)^{c}	1.62 (±0.17)^{abc}	2.53 (±0.13)^{bc}	5.83 (±0.25)^{a}	16.74 (±0.02)^{c}
	H2 (P2 × P3)	0.82 (±0.07)^{b}	14.32 (±0.36)^{a}	44.87 (±1.40)^{d}	6.67 (±0.70)^{b}	33.32 (±2.04)^{c}	2.01 (±0.31)^{ab}	3.30 (±0.70)^{bc}	3.59 (±0.24)^{d}	16.78 (±0.03)^{c}
	H3 (P1 × P2)	0.80 (±0.04)^{b}	9.91 (±0.19)^{c}	40.93 (±0.29)^{fg}	7.55 (±0.07)^{ab}	40.81 (±0.22)^{ab}	1.15 (±0.36)^{c}	3.27 (±0.35)^{bc}	4.48 (±0.03)^{bc}	16.95 (±0.01)^{b}
	T1 (H1 × P2)	0.59 (±0.01)^{c}	9.98 (±0.19)^{c}	43.70 (±0.11)^{de}	6.54 (±0.59)^{bc}	39.19 (±0.51)^{bc}	1.76 (±0.29)^{abc}	3.35 (±0.68)^{bc}	4.85 (±0.15)^{b}	16.90 (±0.01)^{b}
	T2 (T1 segregant)	1.32 (±0.02)^{a}	11.88 (±0.40)^{b}	48.23 (±0.70)^{c}	7.66 (±1.01)^{ab}	30.91 (±2.03)^{c}	2.09 (±0.42)^{a}	2.31 (±0.34)^{c}	3.81 (±0.33)^{cd}	16.75 (±0.02)^{c}
20 °C, 0% EtOH	P1	1.43 (±0.18)^{bc}	25.22 (±0.46)^{a}	32.01 (±0.51)^{d}	15.36 (±0.50)^{a}	25.98 (±0.72)^{cd}	2.52 (±0.13)^{ab}	6.24 (±1.45)^{abc}	1.38 (±0.05)^{d}	16.80 (±0.01)^{bc}
	P2	1.23 (±0.06)^{cd}	16.2 (±0.29)^{e}	37.87 (±0.58)^{bc}	11.04 (±0.51)^{b}	33.67 (±0.37)^{a}	0.74 (±0.46)^{cd}	6.02 (±0.33)^{abc}	2.51 (±0.07)^{a}	16.87 (±0.01)^{a}
	P3	0.91 (±0.03)^{d}	19.68 (±0.59)^{c}	44.57 (±1.22)^{a}	9.25 (±0.81)^{b}	25.57 (±0.57)^{d}	1.44 (±0.51)^{bcd}	5.57 (±0.18)^{bc}	2.35 (±0.08)^{ab}	16.68 (±0.02)^{e}
	H1 (P1 × P3)	0.96 (±0.02)^{d}	19.89 (±0.23)^{c}	40.38 (±1.02)^{b}	11.35 (±0.61)^{b}	27.41 (±0.61)^{bcd}	2.66 (±0.55)^{ab}	6.57 (±0.64)^{abc}	2.11 (±0.08)^{bc}	16.76 (±0.02)^{cd}
	H2 (P2 × P3)	1.33 (±0.14)^{bcd}	22.48 (±0.54)^{bcd}	37.39 (±1.41)^{c}	10.39 (±1.70)^{b}	28.41 (±0.88)^{b}	2.80 (±0.99)^{ab}	7.20 (±1.20)^{ab}	1.93 (±0.18)^{c}	16.75 (±0.02)^{d}
	H3 (P1 × P2)	1.69 (±0.21)^{b}	24.18 (±0.23)^{a}	32.48 (±0.06)^{d}	14.85 (±0.27)^{a}	26.8 (±0.39)^{bcd}	3.63 (±0.48)^{a}	7.17 (±0.60)^{ab}	1.46 (±0.02)^{d}	16.80 (±0.01)^{bc}
	T1 (H1 × P2)	1.06 (±0.13)^{cd}	18.08 (±0.18)^{d}	38.11 (±0.41)^{bc}	10.59 (±0.39)^{b}	32.16 (±0.55)^{a}	0.28 (±0.23)^{d}	8.05 (±0.86)^{a}	2.36 (±0.02)^{a}	16.83 (±0.01)^{ab}
	T2 (T1 segregant)	2.27 (±0.27)^{a}	19.17 (±0.45)^{cd}	39.84 (±1.37)^{bc}	10.94 (±0.30)^{b}	27.79 (±1.13)^{bc}	2.16 (±0.62)^{abc}	4.33 (±0.86)^{c}	2.09 (±0.09)^{c}	16.73 (±0.02)^{d}

Table S1 continued.

Strain and condition		C14:0	C16:0	C16:1	C18:0	C18:1	Squalene	Ergosterol	UFA/SFA ratio	Average FA chain length
10 °C, 8% EtOH	P1	1.18 (±0.27)[a]	24.68 (±2.34)[a]	23.06 (±2.36)[e]	25.75 (±2.04)[a]	25.33 (±2.30)[bc]	3.04 (±1.11)[c]	3.70 (±0.43)[a]	0.95 (±0.18)[d]	17.00 (±0.02)[a]
	P2	0.97 (±0.06)[a]	11.47 (±1.49)[d]	43.68 (±2.24)[a]	13.34 (±1.65)[d]	30.55 (±1.00)[a]	10.94 (±1.68)[a]	1.15 (±0.15)[c]	2.92 (±0.45)[a]	16.86 (±0.01)[b]
	P3	0.97 (±0.07)[a]	18.64 (±0.82)[bc]	42.50 (±0.68)[ab]	15.25 (±0.75)[cd]	22.64 (±0.87)[c]	5.35 (±0.2)[bc]	1.05 (±0.13)[c]	1.87 (±0.12)[bc]	16.74 (±0.01)[c]
	H1 (P1 × P3)	0.69 (±0.17)[a]	16.01 (±0.67)[c]	37.90 (±1.64)[bc]	14.46 (±0.99)[cd]	30.95 (±0.78)[a]	6.30 (±1.55)[b]	2.13 (±0.49)[b]	2.21 (±0.16)[b]	16.89 (±0.03)[b]
	H2 (P2 × P3)	1.05 (±0.21)[a]	19.62 (±1.47)[bc]	34.25 (±2.37)[c]	18.04 (±1.43)[bc]	27.05 (±0.66)[ab]	6.91 (±0.86)[b]	1.09 (±0.03)[c]	1.59 (±0.19)[bcd]	16.88 (±0.02)[b]
	H3 (P1 × P2)	0.98 (±0.19)[a]	20.72 (±1.60)[ab]	26.69 (±1.00)[de]	21.77 (±1.32)[ab]	29.84 (±2.16)[a]	4.28 (±0.92)[bc]	2.18 (±0.16)[b]	1.31 (±0.16)[cd]	17.01 (±0.02)[a]
	T1 (H1 × P2)	1.11 (±0.36)[a]	20.62 (±1.40)[ab]	28.96 (±2.03)[d]	20.61 (±1.60)[b]	28.70 (±1.68)[ab]	5.59 (±0.23)[bc]	1.61 (±0.05)[bc]	1.37 (±0.19)[cd]	16.96 (±0.01)[a]
	T2 (T1 segregant)	NA	NA	NA	NA	NA	NA	NA	NA	NA
20 °C, 8% EtOH	P1	0.28 (±0.04)[b]	7.75 (±0.69)[bc]	35.70 (±1.07)[d]	12.21 (±0.89)[bc]	44.06 (±1.97)[ab]	0.28 (±0.03)[c]	8.01 (±0.93)[a]	3.96 (±0.40)[ab]	17.12 (±0.03)[a]
	P2	0.26 (±0.04)[b]	7.75 (±0.20)[bc]	33.39 (±2.83)[d]	13.95 (±0.28)[a]	44.66 (±3.01)[ab]	0.24 (±0.01)[c]	4.32 (±0.72)[bcd]	3.56 (±0.10)[b]	17.17 (±0.06)[a]
	P3	0.39 (±0.06)[a]	9.13 (±0.21)[a]	58.74 (±0.86)[a]	7.19 (±0.67)[e]	24.54 (±1.40)[d]	0.52 (±0.06)[b]	3.73 (±0.24)[cd]	5.00 (±0.33)[ab]	16.63 (±0.02)[d]
	H1 (P1 × P3)	0.33 (±0.03)[ab]	8.05 (±0.33)[abc]	51.25 (±1.29)[b]	7.36 (±0.32)[e]	33.01 (±0.61)[c]	0.68 (±0.09)[a]	4.85 (±0.06)[bc]	4.80 (±0.98)[ab]	16.83 (±0.04)[c]
	H2 (P2 × P3)	0.23 (±0.01)[b]	8.27 (±0.19)[ab]	41.51 (±0.88)[c]	9.89 (±0.31)[d]	40.10 (±0.79)[b]	0.29 (±0.02)[c]	3.28 (±0.39)[cd]	4.44 (±0.14)[ab]	17.00 (±0.02)[b]
	H3 (P1 × P2)	0.25 (±0.01)[b]	6.96 (±0.43)[c]	35.17 (±1.96)[d]	12.97 (±0.87)[ab]	44.65 (±0.66)[ab]	0.27 (±0.01)[c]	5.66 (±0.23)[b]	3.71 (±0.50)[ab]	17.13 (±0.04)[a]
	T1 (H1 × P2)	0.23 (±0.05)[b]	7.99 (±0.26)[abc]	37.14 (±1.42)[cd]	10.74 (±0.53)[cd]	43.89 (±2.25)[ab]	0.62 (±0.08)[ab]	4.36 (±0.26)[bcd]	3.86 (±0.75)[ab]	17.10 (±0.04)[a]
	T2 (T1 segregant)	0.23 (±0.01)[b]	5.14 (±0.15)[d]	36.22 (±0.53)[d]	10.99 (±0.46)[cd]	47.42 (±0.17)[a]	0.26 (±0.01)[c]	2.97 (±0.17)[d]	5.12 (±0.22)[a]	17.16 (±0.01)[a]

Table S2 – Modelled (*A*, μ, λ) growth parameters of the microplate cultivations performed with the three laboratory strains grown in media supplemented with 0.8 mM oleic acid and various concentrations of ethanol. Growth curves are presented in Figure 7 in the main article. The growth curves were modelled using the logistic model in the ‘grofit’ package for R. Values were determined from four independent cultivations (standard deviation in parentheses). Values in the same group (i.e. the same temperature and ethanol concentration) in the same column with different superscript letters (a–f) differ significantly ($p < 0.05$ as determined with one-way ANOVA with Tukey’s post-hoc test) NA: not available.

**Temperature**	**Ethanol concentration**	**Oleic acid concentration**	**Yeast strain**	**μ (OD600 / hour)**	**λ (hours)**	**A (OD600)**
20 °C	1 %	0 mM	WT	0.086 (±0.001)^{c}	4.8 (±0.1)^{e}	1.69 (±0.002)^{a}
			*ole1*Δ	0.080 (±0.001)^{c}	4.4 (±0.1)^{e}	1.68 (±0.002)^{ab}
			*erg4*Δ	0.095 (±0.001)^{c}	8.1 (±0.1)^{d}	1.69 (±0.002)^{a}
		0.8 mM	WT	0.193 (±0.007)^{b}	18.7 (±0.2)^{b}	1.68 (±0.006)^{ab}
			*ole1*Δ	0.226 (±0.008)^{a}	17.4 (±0.1)^{c}	1.67 (±0.005)^{bc}
			*erg4*Δ	0.215 (±0.008)^{a}	19.9 (±0.2)^{a}	1.66 (±0.007)^{c}
	5 %	0 mM	WT	0.071 (±0.001)^{cd}	7.7 (±0.2)^{e}	1.69 (±0.002)^{a}
			*ole1*Δ	0.062 (±0.001)^{d}	6.3 (±0.2)^{f}	1.69 (±0.002)^{ab}
			*erg4*Δ	0.083 (±0.001)^{c}	13.8 (±0.2)^{d}	1.68 (±0.002)^{ab}
		0.8 mM	WT	0.186 (±0.006)^{a}	23.4 (±0.2)^{b}	1.67 (±0.006)^{b}
			*ole1*Δ	0.189 (±0.006)^{a}	22.6 (±0.2)^{c}	1.65 (±0.006)^{c}
			*erg4*Δ	0.160 (±0.004)^{b}	24.5 (±0.2)^{a}	1.67 (±0.006)^{ab}
	10 %	0 mM	WT	0.043 (±0.001)^{c}	16.2 (±0.4)^{f}	1.73 (±0.005)^{a}
			*ole1*Δ	0.045 (±0.001)^{c}	17.4 (±0.3)^{e}	1.72 (±0.004)^{a}
			*erg4*Δ	0.041 (±0.001)^{c}	21.7 (±0.4)^{d}	1.69 (±0.006)^{b}
		0.8 mM	WT	0.121 (±0.003)^{a}	43.4 (±0.2)^{b}	1.69 (±0.005)^{b}
			*ole1*Δ	0.119 (±0.003)^{a}	38.0 (±0.2)^{c}	1.69 (±0.007)^{b}
			*erg4*Δ	0.098 (±0.003)^{b}	46.3 (±0.3)^{a}	1.66 (±0.008)^{c}
15 °C	1 %	0 mM	WT	0.045 (±0.001)^{c}	10.4 (±0.3)^{bc}	1.65 (±0.004)^{c}
			*ole1*Δ	0.044 (±0.001)^{c}	9.9 (±0.3)^{c}	1.66 (±0.004)^{b}
			*erg4*Δ	0.046 (±0.001)^{c}	11.1 (±0.2)^{b}	1.70 (±0.002)^{a}
		0.8 mM	WT	0.092 (±0.002)^{b}	29.4 (±0.3)^{a}	1.69 (±0.005)^{a}
			*ole1*Δ	0.108 (±0.003)^{a}	28.4 (±0.3)^{a}	1.71 (±0.005)^{a}
			*erg4*Δ	0.106 (±0.002)^{a}	28.5 (±0.2)^{a}	1.70 (±0.004)^{a}
	5 %	0 mM	WT	0.045 (±0.001)^{d}	21.3 (±0.2)^{d}	1.72 (±0.004)^{b}
			*ole1*Δ	0.048 (±0.001)^{cd}	21.8 (±0.2)^{d}	1.72 (±0.004)^{b}
			*erg4*Δ	0.050 (±0.001)^{c}	24.4 (±0.2)^{c}	1.72 (±0.003)^{b}
		0.8 mM	WT	0.071 (±0.001)^{b}	32.2 (±0.2)^{b}	1.74 (±0.004)^{a}
			*ole1*Δ	0.093 (±0.002)^{a}	34.1 (±0.3)^{a}	1.72 (±0.005)^{b}
			*erg4*Δ	0.075 (±0.001)^{b}	31.3 (±0.2)^{b}	1.72 (±0.004)^{b}
	10 %	0 mM	WT	0.026 (±0.001)^{c}	44.5 (±0.5)^{c}	1.78 (±0.014)^{a}
			*ole1*Δ	0.027 (±0.001)^{c}	40.9 (±0.5)^{d}	1.75 (±0.012)^{a}
			*erg4*Δ	0.026 (±0.001)^{c}	44.9 (±0.5)^{c}	1.79 (±0.015)^{a}
		0.8 mM	WT	0.042 (±0.001)^{b}	64.4 (±0.4)^{a}	1.69 (±0.010)^{b}
			*ole1*Δ	0.046 (±0.001)^{a}	60.2 (±0.4)^{b}	1.68 (±0.010)^{b}
			*erg4*Δ	0.045 (±0.001)^{a}	62.9 (±0.4)^{a}	1.67 (±0.009)^{b}

Table S3 – The alleles of the *PAD1* and *FDC1* genes (responsible for the 'phenolic off-flavour'-phenotype) that were detected in the 8 brewing strains based on single nucleotide polymorphisms. Sequences were compared to those of *S. cerevisiae* S288c retrieved from SGD.

**Strain**	***PAD1***	***FDC1***
P1	112C>T, 140C>T, 177C>T, 252C>A, 354T>C, 447C>T, 609G>T	219G>A, 909C>S*
P2	112C>T, 140C>T, 177C>T, 252C>A, 354T>C, 447C>T, 550C>G, 609G>T, 638A>G	219G>A, 339C>T, 673G>A, 1497G>A
P3	159 SNPs	269 SNPs
H1	112C>T, 140C>T, 177C>T, 252C>A, 354T>C, 447C>T, 609G>T	219G>A, 909C>S*
	159 SNPs	269 SNPs
H2	112C>T, 140C>T, 177C>T, 252C>A, 354T>C, 447C>T, 550C>G, 609G>T, 638A>G	219G>A, 339C>T, 673G>A, 1497G>A
	159 SNPs	269 SNPs
H3	112C>T, 140C>T, 177C>T, 252C>A, 354T>C, 447C>T, 609G>T	219G>A, 909C>S*
	112C>T, 140C>T, 177C>T, 252C>A, 354T>C, 447C>T, 550C>G, 609G>T, 638A>G	219G>A, 339C>T, 673G>A, 1497G>A
T1	112C>T, 140C>T, 177C>T, 252C>A, 354T>C, 447C>T, 609G>T	219G>A, 909C>G
	112C>T, 140C>T, 177C>T, 252C>A, 354T>C, 447C>T, 550C>G, 609G>T, 638A>G	219G>A, 339C>T, 673G>A, 1497G>A
	159 SNPs	269 SNPs
T2	112C>T, 140C>T, 177C>T, 252C>A, 354T>C, 447C>T, 550C>G, 609G>T, 638A>G	219G>A, 339C>T, 673G>A, 1497G>A

* Heterozygous SNP

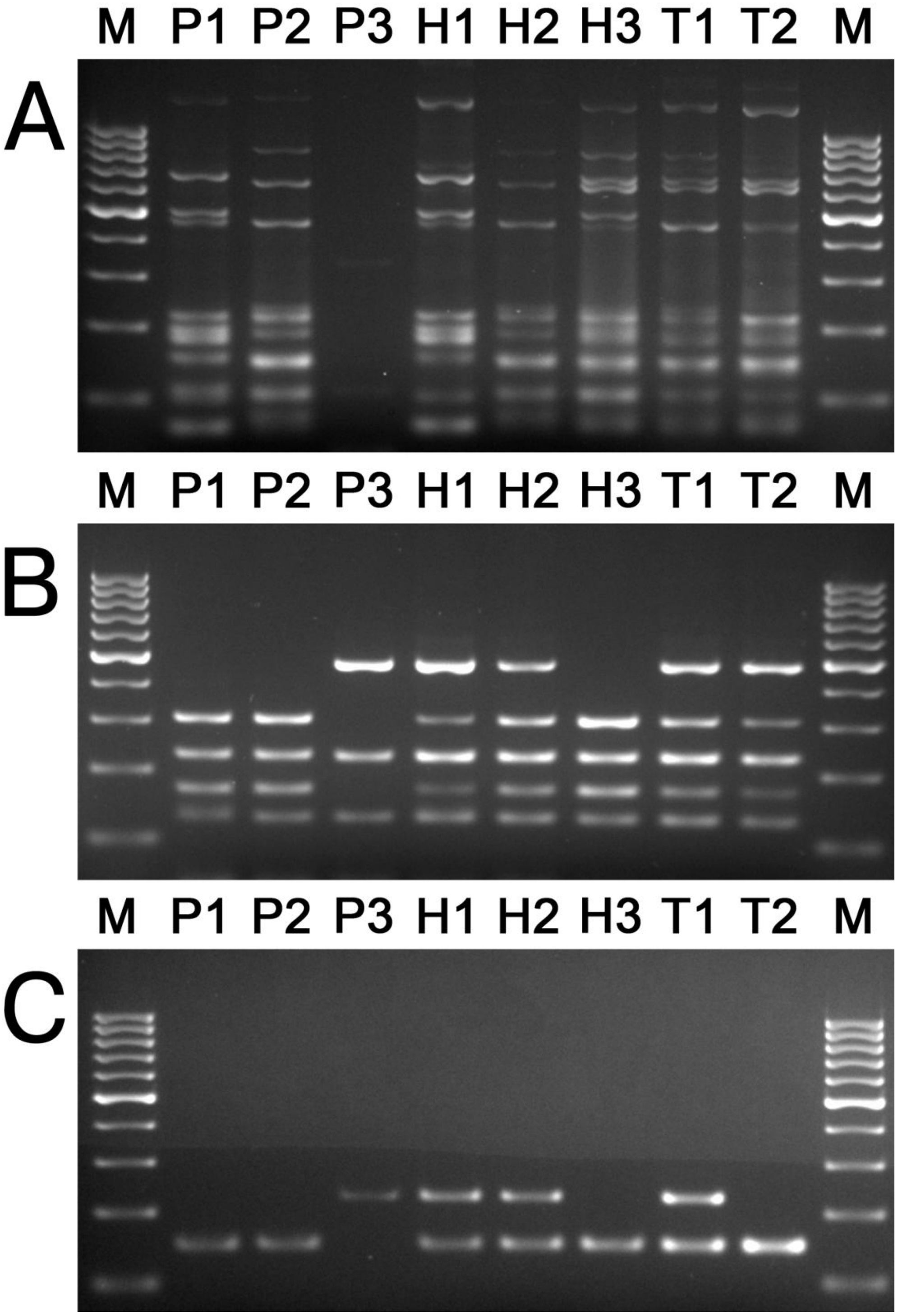

Figure S1 – Confirmation of hybridization by (**A**) interdelta PCR, (**B**) rDNA ITS PCR and RFLP, and (**C**) amplification of *FSY1* and *MEX67* genes using species-specific primers. Lanes M are a 100 bp DNA ladder. As can be seen from (**B**) and (**C**), Hybrids H1, H2, T1 and T2 all contain DNA from both *S. cerevisiae* and *S. eubayanus*. Hybrid T2 has apparently lost the *S. eubayanus*-specific *FSY1* gene through meiotic recombination (**C**). Strains P1 and P2 produced similar band patterns with the interdelta primers, but Hybrids H3, T1 and T2 appear to contain DNA from both P1 and P2 (**A**). This is especially evident in the two bands with a length of approximately 650 bp.

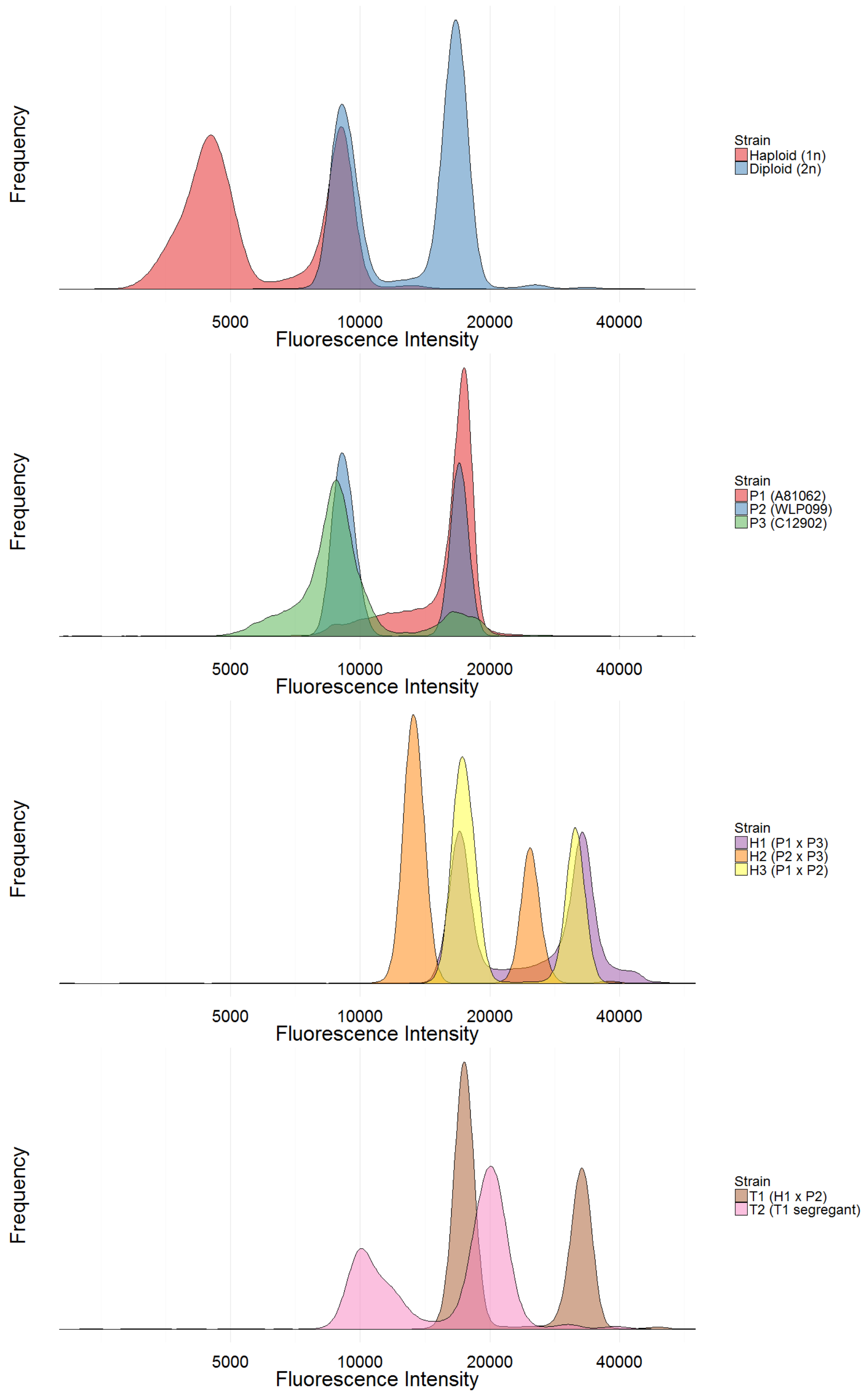

Figure S2 – DNA content of the (**A**) *S. cerevisiae* haploid (CEN.PK113-1A) and diploid (CEN.PK) reference strains, (**B**) P1-P3 parent strains (all diploid), (**C**) H1-H3 hybrid strains (allotetraploid, allotriploid and allotetraploid, respectively), and (**D**) T1-T2 hybrids strains (allotetraploid and allodiploid, respectively) by flow cytometry.

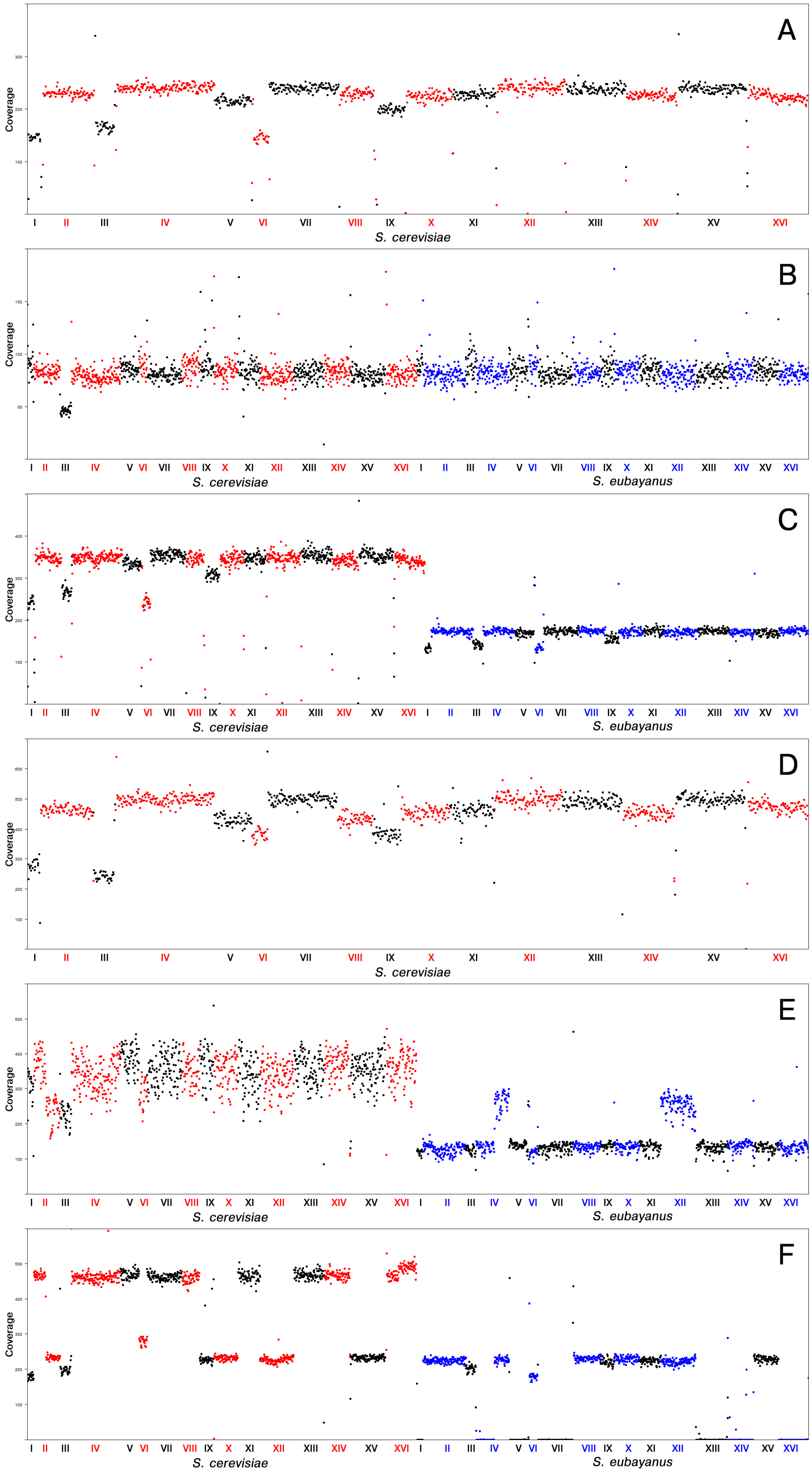
A
Coverage
I II III IV V VI VII VIII IX X XI XII XIII XIV XV XVI
S. cerevisiae
B
Coverage
I II III IV V VI VII VIII IX X XI XII XIII XIV XV XVI I II III IV V VI VII VIII IX X XI XII XIII XIV XV XVI
S. cerevisiae
S. eubayanus
C
Coverage
I II III IV V VI VII VIII IX X XI XII XIII XIV XV XVI I II III IV V VI VII VIII IX X XI XII XIII XIV XV XVI
S. cerevisiae
S. eubayanus
D
Coverage
I II III IV V VI VII VIII IX X XI XII XIII XIV XV XVI
S. cerevisiae
E
Coverage
I II III IV V VI VII VIII IX X XI XII XIII XIV XV XVI I II III IV V VI VII VIII IX X XI XII XIII XIV XV XVI
S. cerevisiae
S. eubayanus
F
Coverage
I II III IV V VI VII VIII IX X XI XII XIII XIV XV XVI I II III IV V VI VII VIII IX X XI XII XIII XIV XV XVI
S. cerevisiae
S. eubayanus

Figure S3 – The sequencing coverage (median in 10 kbp windows) over the *S. cerevisiae*- (black and red) and *S. eubayanus*-derived (black and blue) chromosomes of parent and hybrid strains (**A**) P2 (265x), (**B**) H1 (87x), (**C**) H2 (304x), (**D**) H3 (275x), (**E**) T1 (295x), and (**F**) T2 (317x). The mean coverage of each sample is listed in parenthesis after the sample name.

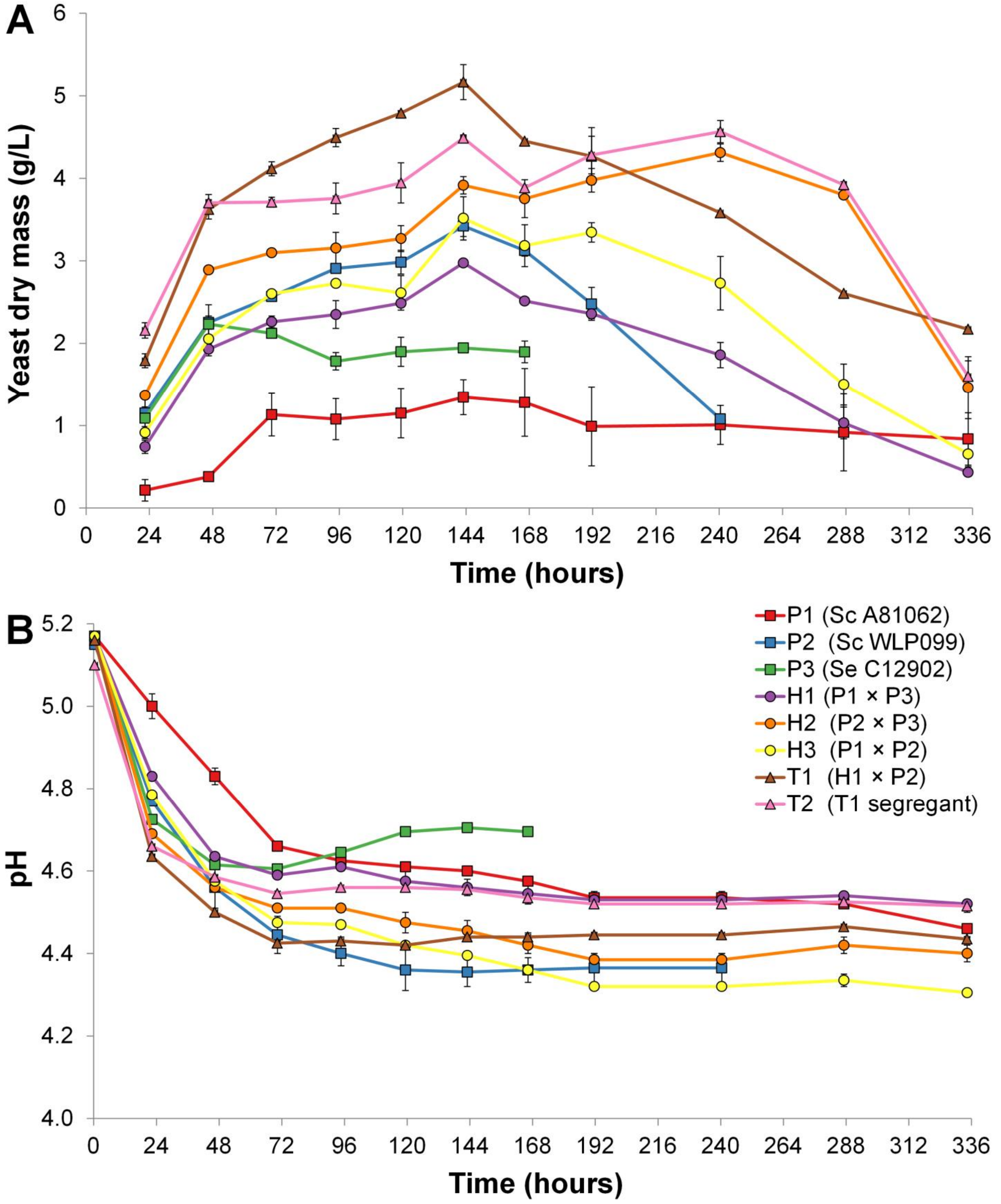

Figure S4 – The (**A**) suspended yeast dry mass (g L^{-1}) and (**B**) pH in the beers fermented from the 15 °P wort with the 8 brewing strains. Values are means from two independent fermentations and error bars where visible represent the standard deviation.
